# Optimal Network for Patients with Severe Mental Illness: A Social Network Analysis

**DOI:** 10.1007/s10488-017-0800-7

**Published:** 2017-03-24

**Authors:** Vincent Lorant, James Nazroo, Pablo Nicaise, The Title107 Study Group

**Affiliations:** 1 Institute of Health and Society, Université Catholique de Louvain, Clos chapelle aux champs 30.15/05, 1200 Bruxelles, Belgium; 2 Cathie Marsh Institute for Social Research, University of Manchester, Humanities Bridgeford Street Building, Manchester, M13 9PL UK

**Keywords:** Referral network, Patients with psychiatric disorders, Optimal structure

## Abstract

It is still unclear what the optimal structure of mental health care networks should be. We examine whether certain types of network structure have been associated with improved continuity of care and greater social integration. A social network survey was carried out, covering 954 patients across 19 mental health networks in Belgium in 2014. We found continuity of care to be associated with large, centralized, and homophilous networks, whereas social integration was associated with smaller, centralized, and heterophilous networks. Two important goals of mental health service provision, continuity of care and social integration, are associated with different types of network. Further research is needed to ascertain the direction of this association.

**Electronic supplementary material:**

The online version of this article (doi:10.1007/s10488-017-0800-7) contains supplementary material, which is available to authorized users.

## Introduction

Continuity of care for patients with severe mental disorders (hereafter, SMI) remains a challenge in many Western countries. Rehospitalization soon after discharge is common, with one study reporting that 12% of patients discharged with schizophrenia are readmitted to the same hospital within the first month after discharge (Pfiffner et al. 2014), as are lack of drug compliance (Fontanella et al. 2014) and the risk of suicide (Qin and Nordentoft 2005). The needs of these patients, moreover, are often not fully met by social services, including those that foster employability (OECD 2013), and they often end-up on long-term sickness leave rather than in employment (Marwaha et al. 2013). There is evidence to suggest that community mental health teams have been able to decrease hospitalization and reduce suicide, perhaps by maintaining continuity of care, but their impact on the use of social services and social integration more broadly is less clear (Malone et al. 2007).

Although coordination is a key component of continuity of care for patients with severe mental disorders (Haggerty et al. 2003), it is often lacking within mental-health services; for example, coordination following hospital discharge is low among patients with schizophrenia (Fontanella et al. 2014). There is also a lack of coordination between primary care and mental health services (Belling et al. 2011; Scharf et al. 2013) and between health care and social care services (Belling et al. 2011; Nicaise et al. 2013; Priebe et al. 2012a, b).

Health care networks have been promoted as a solution that improves coordination across services. These networks take different approaches, depending on national regulations and health care governance (Mitchell and Shortell 2000; Shortell et al. 2014). Broadly, however, they consist of long-term agreements across local organizations in order to provide a local population with a comprehensive range of coordinated (mental) health services aimed at improving community health. In the domain of mental health, the ACCESS program for mentally ill homeless people (Rosenheck et al. 1998) and the Robert Wood Johnson Foundation Program on Chronic Mental Illness (Lehman et al. 1994; Morrissey et al. 1994) are examples of networks that aim to provide continuity of care for vulnerable groups with mental illnesses.

However, the best way to design and implement such networks remains a matter of controversy. Two issues remain unresolved in relation to composition and structure, namely the benefits of a homogeneous network and the benefits of centrally coordinated networks. It is argued by some that effective collaboration across a network is facilitated when it is composed of a limited number of homogeneous members, because too much diversity in a network can lead to discord and hamper reaching agreement on common goals (Mitchell and Shortell 2000). It is also argued that a limited representation of different types of service providers within a network makes it less credible and legitimate (Shortell et al. 2002). Indeed, it is common for a patient with severe mental illness to have contact with a psychiatric hospital outpatient clinic, a community mental health center, an addiction or substance abuse service, a mental health clinician, a crisis center, an assertive community team, sheltered housing, a general practitioner, a social worker, and a job service (Narrow et al. 2000). Hence, networks with a greater diversity of types of service may perform better in relation to patient continuity of care and social integration: they deliver a wider spectrum of services to meet patient needs, they provide better support for maintaining the patient in the community, and they are more responsive to patient preferences. So far, however, the evidence is not conclusive on how a network’s size and composition relate to its effectiveness at the client level (Turrini et al. 2010).

The coordination structure of complex networks remains another contentious topic. For the sake of simplicity, a network can be coordinated either by one agency taking up a central role, or by supporting a dense network of ties between all agencies (Morrissey et al. 1994). It has been suggested that effectiveness at the patient level is increased when coordinated by a central agency, rather than when all agencies take it upon themselves to integrate their services, in both the domain of mental health (Provan and Milward 1995) and the domain of general health (Chukmaitov et al. 2009; Mascia et al. 2015). Indeed, as far as patients with a severe mental illness are concerned, coordination by a central agency makes more sense: their multiple social vulnerability weakens their capacity to navigate complex mental health and social systems and a central organization is needed to take over the role of coordinating the different services in order to avoid hospitalization or other adverse events (Leutz 1999). However, more centralized networks may struggle to deliver a broader range of services, which, in turn, may limit patient continuity of care (Bazzoli et al. 1999). In centralized networks, moreover, psychiatric hospitals are more likely to take up that coordinating role, given their financial, staffing, and logistical resources, and this may conflict with the overall aim of moving the delivery of services into the community and improving patients’ social integration (Nicaise et al. 2014).

So it seems that SMI patients need “impossible” networks, delivering both differentiation at the level of service delivery and centralization at the level of network governance. Bazzoli and colleagues indicated that these two network features were at odds with each other (Bazzoli et al. 1999). In this inconclusive, paradoxical, context, the promotion of mental health networks by public authorities or mental health agencies has relied on very limited empirical evidence about the most effective network structure for patient outcomes (Provan et al. 2007; Provan and Milward 2001). It is unfortunate that so little empirical research has been done to assess which network structure is most suitable for patients with severe mental illness, particularly given the severe limitations networks may have (McGuire and Agranoff 2011). In the domain of mental health, there are only a few studies, most of them in the U.S., and with a limited number of networks and small sample sizes (Provan and Milward 1995; Provan and Sebastian 1998). Beyond the available taxonomies of networks (Bazzoli et al. 1999; Shortell et al. 2014), we need a broader empirical basis to analyze the relationship between network structure and effectiveness for patient-level outcomes such as continuity of care, quality of life, social integration, and recovery (McGuire and Agranoff 2011).

In line with theory on inter-organizational networks and with reviews of network effectiveness (Leutz 1999; Provan and Milward 1995; Provan et al. 2005; Turrini et al. 2010; Nicaise et al. 2013), we hypothesized that continuity of care and social integration would be improved by differentiated networks, by integrated networks, and by heterophilous networks (that is, involving ties to other types of services). Service networks are differentiated when they include a broad range of service types, i.e. delivering different kinds of interventions, e.g. outpatient mental-health care, social care, long-term housing, etc. (Bazzoli et al. 1999). Networks are integrated when there are tight referral relationships within the network. Integration can be achieved, for example, with a high level of connectivity, or with a strong leadership. Heterophily in a network is the tendency of services to have connections with services of a different type (in contrast to homophily). Yet, as has been shown in the past, these characteristics may not be compatible with each other. For example, a network may not achieve both differentiation and integration at the same time (Morrissey et al. 1994).

In this context, this paper addresses three research questions:

- What are the structural features of networks of referrals between services addressing the needs of patients with severe mental illness?
- What network structure and network composition is most suitable for continuity of care for patients with severe and chronic mental illness?
- What network structure and network composition is most suitable for the social integration of these patients? And, consequently, can a single network structure address both issues?

## Method

### Setting

This research is part of a wider evaluation of the Belgian Mental Health Reform, which has been described elsewhere (Nicaise et al. 2014; Lorant et al. 2015). The reform was based on the establishment of networks of (mental) health and social services which are intended to supply comprehensive care to all adult mental health users. The main goals of the reform were (1) to reduce the frequency and duration of psychiatric hospitalization, (2) to strengthen community-based mental health care delivery; (3) to improve continuity of care; and (4) to improve the social integration of patients with psychological problems. To achieve these overarching goals, networks of mental health services are requested to provide five basic care functionalities for all adult patients located in a catchment area: (1) prevention and early detection of mental illnesses (primary care and community mental health services), (2) crisis and outreach services, (3) rehabilitation (rehabilitation teams and social services), (4) intensive residential treatment for acute cases (psychiatric wards), and (5) long-term care and housing facilities (sheltered housing and psychiatric nursing homes).

### Design

We carried out a social network survey with information collected at the level of the services involved in these networks and at the level of the patients cared for by the participating services. Social network analysis is a method of data collection and analysis of the structure of connections between actors (Provan et al. 2005) and has four cornerstone components: the network boundary (who is in and who is out), the nodes (i.e. organizations), the ties (i.e. exchange links), and the data collection method (Knoke et al. 2008). All the 19 networks of mental health services funded by the public authorities, in 2012 and in 2014, were included. These networks covered most metropolitan areas of the country, north and south, as well as some rural areas. The areas covered differed greatly in terms of deprivation and population density, with more deprived areas in the south compared with the north. Population density was generally quite high, as most projects targeted urban areas, but three networks were implemented in rural areas. Nodes were agencies who were members of the network, regardless of the type of mental, general health, or social care they provide. Ties were the clinical and organizational activity links between these agencies. In addition to the services data, a sample of patients was selected within each network. Data collection was carried out in 2014, using a web survey for agency information and a paper questionnaire for patients’ data.

### Data Collection

In each network, all mental, social, and general health services were invited to participate in two data collections, at the service level and at the patient level. First, each service filled out an online questionnaire recording links with other services within the same network. The links connecting services were identified on the base of (1) referrals to other services, (2) referrals received from other services, (3) information exchanges related to the patient, and (4) organizational activities, in a way similar to previous studies (Morrissey et al. 1994; Milward and Provan 1998; Provan and Sebastian 1998; Provan et al. 2005; Nicaise et al. 2013). Data were collected by a one-mode design: each service received a complete list of network members and rated the frequency of contacts with the other services during the previous 6 months: never, sometimes, or often. In the adjacency matrix, the ordinal values were dichotomized: “sometimes” and “often” were categorized as 1, “never” as 0. Services were requested to fill out the questionnaire during staff meetings, so as to gather all the information required from the different staff roles (clinicians and managers). The questionnaire was filled out by managers and heads of services (54%), social work or administrative staff (18%), health professionals (13%), and other staff (16%).

Second, 80 patients with severe mental illness (SMI) were sampled in each network. SMI was defined as meaning patients with a psychiatric diagnosis, who had been in contact with psychiatric services for at least 2 years, had experienced at least one hospitalization, and had disability in a social role (Schinnar et al. 1990). Eligible patients were identified at the service level by the clinicians. Cluster sampling was applied and ten patients were selected in each of the following eight services, in order to capture the diversity of services and patients: two services from primary care and community mental health, two services from crisis/outreach teams, two psychiatric wards, one long-term care residential service (either sheltered housing or a nursing home), and one social or rehabilitation service. Within each service, patients were selected by systematic sampling from the admission directory or resident directory: each service selected every pth patient on an alphabetically sorted list where p was the number of eligible patients divided by 10. Exclusion criteria were: the patient was unable to consent, or the patient was unable to fill out a questionnaire in one of the national languages. Ethical approval was obtained on 31 March 2014, from the Ethical Committee of KU Leuven Medical Centre under the reference no. B322201215190 - study no. S54355.

### Exposure Measures at the Network Level

Firstly, we hypothesized that continuity of care and social integration were associated with network differentiation. Differentiation was measured by the size of the network (number of services) and by the composition of networks in terms of proportions of service types. We also computed the index of dissimilarity (ID), which is a measure of uneven distribution of the different type of services; it measures the departure from the average distribution of the different types of service as a whole. The ID index is commonly used in social epidemiology (Oakes and Kaufman 2006): a value of 0 means the distribution of services in a network is equal to the average distribution of services in all networks, a value of 0.5 means that across all type of services there is an excess of 50% in the percentage of one type of service compared with the average distribution across all networks.

Secondly, we hypothesized that continuity of care and social integration were associated with network integration. The integration of the network structure was assessed using three indices: density, centralization, and clustering. The first, density of ties, captures the cohesion of the network and is the ratio between the number of ties reported and the number of possible ties. The second, degree centralization, is a measure of how unequal the services are in terms of the number of their ties. Degree centralization ranges from 0 to 1:0 means that all services have an equal number of ties, while 1 means that one service is the only one connected with all the other services in the network. The last one, clustering, is a measure of cohesion around a service and is calculated as the density of connections around that service. At the network level, it takes the value of 0 if none of the neighbors of a service are connected to each other, and a value of 1 if all those neighbors are connected to each other.

Thirdly, we hypothesized that continuity of care and social integration were associated with referral heterophily, that is a referral to a different type of service. The availability of weak ties in heterophily was assessed by the Coleman heterophily index, an index which captures network heterophily both at the network and node levels, is applicable to directed networks, and takes the value of zero when the referral distribution between and within types of services matches the marginal distribution of the type of services (random network property) (Bojanowski and Corten 2014). For each type of service, we computed the Coleman index, which ranges from −1 for perfect heterophily (all referrals are to a different type of service) to +1 for perfect homophily (all referrals are to the same type of service), with 0 meaning the referrals are distributed consistently with a random network.

### Patient Outcomes: Continuity of Care and Social Integration

The reform program considered two main patient-level outcomes: experienced continuity of care and social integration. Experienced continuity of care was measured using the Alberta Continuity of Service Scale for Mental Health (ACSS-MH) (Adair et al. 2003, 2005; Joyce et al. 2010). This is a 31-item scale that captures how the patient experiences continuity of care in three main dimensions: individualized care, system responsiveness, and carer responsiveness. Each item ranges from “1-completely disagree” to “5-completely agree” and the total score has a maximum value of 155. Social integration was measured using the SIX index, a measure of social integration suited to long-term psychiatric adult patients. The SIX index is a simple, meaningful score that summarizes indicators of social outcomes in mental health care and covers four main dimensions of social integration: employment, accommodation, family relationships, and friendship (Priebe et al. 2008). The SIX returns a score ranging from 0 (no social integration) to 6 (high social integration).

### Clinical and Socio-Demographic Information

In addition, each patient’s clinician provided clinical information, including the main diagnosis (DSM-IV) and the extent to which the patient’s psychosocial functioning was impaired, using the Health of the Nation Outcome Scale (HoNOS), which ranges from 0 (no impairment) to 48 (extreme impairment) (Wing et al. 1998). Additional socio-demographic information was requested from both the patient and the clinician.

### Statistical Analysis

We first tabulated socio-demographic and clinical characteristics, the different items related to continuity of care and the social integration of patients. Second, we described the network structures and computed Pearson correlation coefficients to examine the relationships between different metrics of network structure. In the third step, we used regression analysis to relate network metrics with patient outcomes. We used linear regression for continuity of care and multinomial regression for social integration. As we included only 19 networks, collinearity between the different dimensions of the network structures is likely, so we carried out first bivariate regressions to select the most significant covariates for the multivariate analysis. In the first model, we carried out bivariate analysis, controlling for confounders; in the second model, we carried out stepwise multivariate analysis with all variables that were statistically significant in the previous step. The association between network exposure and continuity of care was controlled for patient-level variables that might influence both patient selection and continuity of care. In the light of previous studies (Chukmaitov et al. 2009; Mascia et al. 2015), we controlled for age, sex, HoNOS score, and the type of service through which the patient was contacted.

Finally, two sensitivity analyses were carried out. First, the models were replicated with the full-network matrices, whether the services had participated or not. This would help us to assess the impact of services participation; second, the adjacency matrices were recomputed with only “often” categorized as 1 and “never” and “sometimes” both categorized as 0.

## Results

Of the 991 services registered in the 19 networks, 518 completed the survey (participation rate = 52%). At the patient level, 1078 patients completed the survey out of the 1520 invited, giving a participation rate of 71%. After excluding missing items, we were left with 954 complete records.

### Patient-Level Results

The average continuity of care was good, with a score of 115.6 (std = 14.1) out of a maximum of 155 (Table 1). However, items related to treatment responsiveness received lower scores (mean = 70.7%), whereas items related to relational continuity had the highest scores (mean = 77.7%). Topics where levels of satisfaction were lower involved: patients reporting having to repeat their history each time they needed help, the primary clinician not checking on patients, the mental health professional not being in touch with the GP, the patient being unable to get services in the middle of the night, the patient not knowing the services available, and providers not having the patient’s records when needed (see Supplementary Table 1). Over the last 6 months, patients had 1.5 outpatient contacts, 0.7 contacts with a social service, and 0.7 hospitalizations or long-term stays. Patients had a low level of social integration, with an average score of 3.1 (std = 1.3) out of a maximum of 6.

**Table 1:** Patient characteristics, outcomes, and use of services: mean and std: study of Belgian mental health networks, 2014 (n = 954)

Variable	Mean	Std
Patient outcomes		
Alberta continuity of care (31–155)	115.6	14.1
Social integration (SIX score, 0–6)	3.1	1.3
Use of services		
Outpatient services (no.)	1.5	1.2
Social services (no.)	0.7	0.9
Residential services (no.)	0.7	0.7
Socio-demographics and clinical status		
Age (y)	45.7	12.6
Male (%)	48.0	0.5
Global HoNOS score (/48)	12.5	6.5
Principal diagnosis (%)		
Schizophrenia – other psychotic disorder	28.6	
Mood disorder	25.4	
Substance use	17.4	
Personality disorder	15.1	
Anxiety disorder	6.5	
Other – non specified	7.0	

### Network-Level Results

The 19 networks are described in Table 2. Overall, the networks displayed great diversity of composition. They were composed of 51.5 services on average: the smallest network included 11 services, while the largest included 115 services. Networks were composed mainly of social services (20.9%) and psychiatric wards (17.1%). Community mental health teams (10.8%) and primary care services (12.8%), the services which should provide alternatives to hospitalization, were among the least frequently reported services in these networks. The composition of these networks departed from the average distribution by 25.4%, suggesting that on average there was a good balance in the composition of the networks. The networks were also well integrated, with a high density of connections between services (48.9%) and a lower degree of centralization (24.8%). The clustering coefficient (64.1%) suggested the networks were generally organized around a cohesive sub-group of services. Networks had negative Coleman indices, indicating that all types of services had similar levels of heterophily. As these networks were commissioned at two different moments (2012 and 2014), we compared these network covariates for these two cohorts (see Supplementary Table 2). The networks of the 2012 cohort were bigger (mean size = 59.9) than those of the 2014 networks (mean size = 32.5, *t* test=−2.3, *p* = 0.03) and the Coleman index for crisis/outreach teams was less homophilous (mean Coleman for 2012 cohort = −0.2 vs. mean Coleman for 2014 cohort = −0.5, *t* test=−2.1, *p* = 0.05). No significant differences were noted for the other metrics. This indicates that, over time, a network may tend to expand and that the new (crisis/outreach team) service may require time before connecting to other existing services.

**Table 2:** Network structure descriptive statistics, study of Belgian mental health networks, 2014 (n = 19)

Network structure covariates	Mean	Std	Min	Max
Diversity and composition				
Services (no.)	51.5	31.3	11.0	115.0
Primary care (%)	12.8	7.2	2.0	27.3
Community mental health (%)	10.8	7.5	1.0	32.8
Crisis/outreach team (%)	10.0	3.7	4.9	18.2
Rehabilitation team (%)	11.6	5.6	0.0	25.5
Social services (%)	20.9	16.3	0.0	66.0
Psychiatric wards (%)	17.1	10.0	3.3	34.4
Sheltered housing (%)	10.2	5.9	1.5	23.5
Nursing homes (%)	3.5	3.5	0.0	9.8
Other (%)	3.1	4.6	0.0	16.7
Dissimilarity index (%)	25.4	8.2	13.4	42.2
Integration				
In-degree normalized	11.1	4.6	3.4	21.1
Degree centralization (%)	24.8	19.5	3.2	74.2
Density (%)	48.9	13.7	25.7	74.0
Clustering (%)	64.1	10.0	46.8	83.4
Weak ties				
Coleman index primary care (−1,1)	−0.3	0.4	−1.0	0.2
Coleman social/rehabilitation services (−1,1)	−0.2	0.3	−1.0	0.1
Coleman crisis/outreach teams (−1,1)	−0.3	0.4	−1.0	0.2
Coleman psychiatric wards (−1,1)	−0.3	0.4	−1.0	0.1

The referral networks are displayed in supplementary materials. Graphs are sorted by size. Nodes represent services. Lines represent clinical contacts (referrals sent, referrals received, and information exchange about patients). The size of nodes is proportional to nodes’ in-degree centrality. The shape of nodes represents the type of setting (outpatient health, inpatient health, home treatment, or other – see legend in each graph), and the color distinguishes service types. The graph layout was created using the Kamada–Kawai algorithm.

Table 3 displays the relationships across the different structural features. Most of these structural features were strongly interrelated. Large networks were more centralized (ρ = 0.78), whereas centralized networks were less dense (ρ = −0.62). Dense networks had higher clustering and reciprocity (ρ = 0.78). Interestingly, referral homophily of crisis and outreach teams was positively associated with homophily of social services (ρ = 0.71). Finally, the index of dissimilarity was correlated with none of the previous indicators. A network with a more unequal distribution of the different types of services, however, was more likely to be associated with more heterophilous primary care services (ρ = −0.69). In other terms, primary care services were in contact with more diverse types of services when types of services were more unequally represented within the network.

**Table 3:** Correlation among network structures, study of Belgian mental health networks, 2014 (n = 19)

Network covariate	Pearson correlation coefficient (−1,1)							
	Dissimilarity (%)	Degree centralization (%)	Density (%)	Clustering (%)	Coleman – primary care	Coleman – crisis/outreach	Coleman – social/rehab	Coleman – psychiatric wards
Services (no.)	−0.16	0.78***	−0.68**	−0.49*	−0.17	0.60**	0.55*	0.46*
Dissimilarity index (%)	1.00	−0.05	0.40	0.38	−0.69**	0.35	0.22	−0.06
Degree centralization (%)		1.00	− 0.62**	−0.37	0.14	0.51*	0.58**	0.54*
Density (%)			1.00	0.83***	−0.15	−0.07	−0.24	−0.39
Clustering (%)				1.00	−0.1	0.03	0	−0.21
Coleman index – primary care (−1,1)					1.00	0.06	−0.06	−0.09
Coleman index – crisis/outreach (−1,1)						1.00	0.71***	0.34
Coleman index – social/rehab (−1,1)							1.00	0.44

Correlation significant at: *5%, **1%, ***1‰

Table 4 describes the association between network composition structure and patients’ continuity of care. The total score for continuity of care was not related to the network structure, with the exception of Coleman indices: higher homophily of the referral relation (or lower heterophily) was associated with a slight increase of continuity of care. This association remained significant in the multivariate analysis of the homophily of crisis and outreach teams: the fewer services mobile teams declared referrals to, the higher the score for continuity of care (Std β = 0.08). This would suggest that mobile teams have to have connections with a small number of different services.

**Table 4:** Effect of network structure on patient subjective continuity of care (Alberta CSS), standardized beta coefficients from the regressions, study of Belgian mental health networks, 2014 (n = 954)

Network covariates	Alberta continuity of care: total score				Alberta continuity of care: relational base			
	Models 1^(§,#)^		Model 2^(§,#)^		Models 1^(§,#)^		Model 2^(§,#)^	
	Beta†	*p* value	beta^†^	*p* value	beta^†^	*p* value	beta†	*p* value
Services (no.)	0.06	0.12			0.17	<0.01		
Social services (%)	−0.00	0.91			0.07	0.04	0.08	0.04
Index of dissimilarity (%)	−0.04	0.27			−0.05	0.14		
Degree centralization (%)	0.04	0.24			0.16	<0.01		
Clustering (%)	0.03	0.39			0.00	0.90		
Density (%)	−0.02	0.66			−0.17	<0.01	−0.16	0.03
Coleman index prim. care (−1,1)	0.08	0.02			0.06	0.09		
Coleman index mobile team	0.08	0.02	0.08	0.02	0.09	0.01		
Coleman index social services	0.08	0.02			0.14	<0.01	0.06	0.07
Coleman index psy. wards	0.01	0.79			0.12	0.01		
Intra-class correlation network-level (%)	3.2% (*p* = 0.06)^a^		3.5% (*p* = 0.07)		6.21% (*p* = 0.02)^a^		3.7% (*p* = 0.09)	
Akaike information criteria	6987.2		6844.8		4608.9		4355.7	

^§^All models are controlled for patient age (y.), sex, HONOS score, and type of service where the patient was recruited

^#^Model 1 adds each network covariate in the regression separately; Model 2 is a stepwise regression of the network covariates significant in Model 1

^†^Standardized betas

^a^For Model 1, intra-class correlation and AIC correspond to the first bivariate regression (no. of services)

Relational-based continuity of care was higher when a network included a greater number of services, particularly when the network had a higher proportion of social services. As structural features were strongly correlated, relational continuity of care was also higher when the network was more centralized (Std β = 0.16), was less dense (Std β = −0.17), and had greater referral homophily. In the multivariate model, the increase of relational continuity of care was still associated with the proportion of social services, the density of the network, and the homophily of social services (with borderline statistical significance).

Finally, Table 5 investigates the association between network composition and structure and patients’ social integration. In the bivariate analysis, we found that social integration was greater in networks that were less centralized (odds ratio = 0.98), were denser (odds ratio = 1.02), had a smaller number of services (odds ratio = 0.99), and had heterophilous relationships across service types. In the second model, social integration was supported by smaller networks that had less density, were more centralized (but with borderline significance), and had heterophily of social services and primary care services.

**Table 5:** Effect of network structure on patient social integration (SIX score), odds ratio from the multinomial regression, study of Belgian mental health networks, 2014 (n = 954)

Network covariates	Model 1*		Model 2*	
	Odds ratio	*p*	Odds ratio	*p*
Services (no.)	0.99	<0.01	0.97	<0.01
Social services (%)	0.99	0.02	1.01	0.19
Dissimilarity index (%)	0.99	0.37		
Degree centralization (%)	0.98	<0.01	1.02	0.07
Density (%)	1.02	<0.01	0.94	<0.01
Coleman index – primary care (−1,1)	1.66	<0.01	0.17	<0.01
Coleman index – mobile team (−1,1)	0.49	<0.01	11.27	<0.01
Coleman index – social services (−1,1)	0.35	<0.01	0.29	0.04
Intra-class correlation network-level (%)	9.9%	0.01	12%	0.03
Akaike information criteria	2588.8		2450.2	

*Model 1 is bivariate regression; Model 2 is multivariate regression; *controlled for age, sex, HoNOS score, and type of services

Tables 4 and 5 were replicated with the network indicators recomputed with the full-network matrices, whether the services had participated or not. We observed very minimal changes in the coefficients. The only significant change was in the reciprocity coefficient, which became significant for patients’ social integration (OR 1.05, p = 0.03). Finally, the adjacency matrices were redefined to consider only ties categorized as “often”. First, we noted that the absolute value of the correlation coefficients across network covariates decreased slightly. Second, beta coefficients (Table 4) or odds ratios (Table 5) were rather stable: the statistical significance was increased for most coefficients; however, the coefficients related to social services were either smaller or statistically non-significant, possibly because the frequency of contacts between mental health services and social services was no longer captured with this categorization of ties.

## Discussion

So far, there is little empirical basis underpinning the structure of networks for the provision of services for patients with mental illnesses. Our study is among the first to analyze the link between the composition and structure of networks of services and patient outcomes, looking at a broad range of services, including mental services, inpatient and outpatient services, and social and medical care services. We described the structure of 19 newly established networks, as well as outcomes for more than 1000 patients across Belgium. This study yielded two main findings:

1. Some network characteristics conflict with each other. On the one hand, networks have to decide whether they wish to achieve integration through density of contacts or centralization. On the other hand, in larger networks with diverse services, the likelihood that services will have actual contacts with different services is lower. It is thus impossible to design an optimal network for the different needs of patients with severe mental illnesses.
2. Patient-level outcomes of continuity of care and social integration are enhanced by somewhat different network structures. Patients’ social integration benefits from smaller, centralized (or less dense), and heterophilous networks (particularly when they include primary care and social services). The relational dimension of continuity of care benefits from larger, less dense, more centralized, and more homophilous networks. However, global continuity of care is not associated with network structure/composition. Centralization is the only structural feature that enhances both outcomes.

## Consistency and Interpretation

Few studies have empirically examined the optimal structure for mental health and social care services. The landmark study by Bavelas and Levitt used an experimental design to identify the optimal structure for simple communication between individuals (Leavitt 1951). They found that a centralized network structure (i.e. one individual brokering the contacts between all other individuals) was faster, more efficient, and had fewer errors compared with a circle structure (i.e. each individual being equally in-between the others). Later research, however, has shown that the outcome also depends on the task’s complexity: a hierarchical (or centralized) structure was associated with a lower level of performance for more complex tasks (Cummings and Cross 2003). Hence, from a structural point of view, a balance has to be found between centralization and density. In the field of mental health care, Morrissey, and later Provan and Milward, have also shown that density and centralization cannot be maximized simultaneously (Morrissey et al. 1994; Provan and Milward 2001).

The finding that centralization improves patient-level outcomes is also consistent with studies in the domain of general health. For example, hospital systems that are more centralized display better outcomes for some aspects of inpatient care, such as lower mortality rates, than other kinds of hospital systems (Chukmaitov et al. 2009). In another (Italian) study, patient readmission to hospitals (a negative proxy for quality of care) was less likely when the hospital was more central and better connected to other key hospitals (Mascia et al. 2015). However, those studies suggested that network structure has to be adapted to the goal of the network. If social integration is the network priority, then, according to our study, small, centralized, and heterophilous networks may be required. However, if continuity of care is the priority, then larger, centralized, and homophilous networks may be more effective. This is the structure that some mental health and social services trusts have implemented in the UK.

Our results suggest that one single network structure is not adapted to the different goals of the reform, in particular continuity of care and social integration. Continuity of care was associated with large, centralized, and homophilous networks. This is consistent with previous research by Provan and Sebastian (Provan and Sebastian 1998), and to some extent with the reform program, which suggested that services should cluster around five basic care functionalities covering the main patient needs. Indeed, that specific pattern of network structure combines the advantages of a large range of services (size), one actor in a coordinating position (centralization), and intense integration in subgroups of services. Networks P17 and P15 (see graphs in the supplementary material) are good examples of this. On the other hand, social integration was associated with smaller, centralized, and heterophilous networks. Social integration does not require as large panel of different services as continuity of care does; it does, however, require more intense support of the user in everyday life activity. Networks P4, and P5 are good examples of this. Another element that might play a role is the fact that users who require more continuity of care may have less capacity for self-direction in navigating the network (Leutz 1999), whereas those with a higher level of social integration also have greater capacity for self-direction, which is an important element in the Belgian health system, in which users have considerable autonomy when it comes to choosing providers and there are no formal referrals.

In the context of the reform, the former has to do with mechanisms for care coordination, while the latter has to do with social rehabilitation and recovery (Nicaise et al. 2014; Lorant et al. 2015). Hence, the structure of networks should be more explicitly defined at the policy level, depending on priorities. It should also be possible to combine the two types of structure by differentiating the structure of organizational and governance relations from the structure of clinical relations (referrals and information exchanges about patients). A large, centralized organizational network may support smaller, cohesive, and heterophilous sub-networks that may have, at their center, a social service. This would be consistent with the results of Provan and Sebastian 1998 – networks within networks (Provan and Sebastian 1998).

## Limitations

This study has three limitations, namely its design, the lower participation rate in some networks, and the data collection method. Firstly, the cross-sectional design makes it difficult to disentangle two relationships between network structure and patient outcomes: whether the network structure has affected patient outcomes or whether it reflects the type of patients included. Networks were commissioned on the basis of the local composition of services in a catchment area; it is thus possible that patient profiles differed between networks whose structure had been designed according to patients’ needs. In a way, an optimal network can also be expected to have a structure that best suits its target population: networks with higher levels of social deprivation may be those that are able to support patients of this kind and keep them in the health system, as opposed to networks where patients are in a better social situation. Controlling for patient’s HoNOS scores, socio-demographics, and the type of services makes it possible to avoid comparing heterogeneous groups of patients and limits selection effects at the service level, but it does not avoid other selection effects on unobserved factors. Below, we provide suggestions on how to address this issue.

Secondly, some networks had a lower participation rate both for patient and service surveys. This may have led to misclassification of network exposure if participating services had a different structural position within the network compared to non-participating services, in particular for networks with low participation rates. This risk was assessed by sensitivity analysis and overall we found little evidence that non-participation had an effect on the estimates.

Finally, a more generic limitation is related to the specific context of the study, i.e. the characteristics of the Belgian mental-health system. We cannot rule out the possibility that, within a different organizational context, associations between network-level indicators and patient-level indicators would be different. The assessment of networks in terms of structural indicators, however, makes it possible to compare networks of services from a formal point of view, regardless of contextual factors. Hence, this analysis may be replicated in other organizational contexts in future research.

## Directions for Future Research

Two improvements may bring better inference and validity in the future. First, longitudinal data on both patients and network structures will help to disentangle two relationships: (1) how patient social integration and continuity of care are affected by changes in the network structure and (2) how network structure evolves in response to the patients cared for. Secondly, the structure of networks was measured with nomination networks of referrals and information exchange, as a proxy for actual referrals or as a proxy for the organizational structure of the network. Although this approach has been used before, it only imprecisely reflects these network structures. Accurate recording of the real set of referrals and patient care pathways would probably provide more accurate information. This alternative would be made much more feasible by using the information from patient electronic records, now widely available in the health sector. Convincing the social service sector to be involved in this information-sharing may pose practical challenges.

## Conclusion

While networks for SMI patients are bound to include many different services, due to the multiple needs of patients, they are likely to be centralized and more homophilous. This may jeopardize continuity of care, which requires diversity of services. Also, this may put the psychiatric hospital back at the center of mental health care, which conflicts with the aims of many mental health care reform programs. The structural alternative, in order to address this issue, is to develop large, centralized networks of organizational relations which include denser, smaller, and less diverse sub-networks of clinical relations. The large organizational network can be centralized in NAO (Network Administrative Organization) scheme, avoiding the predominance of psychiatric hospitals, and favoring the involvement of primary care and social services. In the absence of a NAO (which is ruled out in the current reform), the second alternative is to switch decision-making powers to social service organizations, a model that favors trust-like organization, as implemented in the UK.

## Electronic Supplementary Material

Below is the link to the electronic supplementary material.

Supplementary material 1 (DOCX 15 KB)

Supplementary material 2 (PDF 3002 KB)

## References

[CR1] Adair CE, McDougall GM, Beckie A, Joyce A, Mitton C, Wild CT, Costigan N (2003). History and measurement of continuity of care in mental health services and evidence of its role in outcomes. Psychiatric Services.

[CR2] Adair CE, McDougall GM, Mitton CR, Joyce AS, Wild TC, Gordon A, Beckie A (2005). Continuity of care and health outcomes among persons with severe mental illness. Psychiatric Services.

[CR3] Administration and policy in mental health and mental health services research. http://dx.doi.org/10.1007/s10488-013-0500-x.

[CR4] Bazzoli GJ, Shortell SM, Dubbs N, Chan C, Kralovec P (1999). A taxonomy of health networks and systems: Bringing order out of chaos. Health Services Research.

[CR5] Belling, R., Whittock, M., McLaren, S., Burns, T., Catty, J., Jones, I. R., & Wykes, T. (2011). Achieving continuity of care: Facilitators and barriers in community mental health teams. *Implementation Science, 6*(1). doi:10.1186/1748-5908-6-23. 10.1186/1748-5908-6-23 PMC3073925 21418579

[CR6] Bojanowski M, Corten R (2014). Measuring segregation in social networks. Social Networks.

[CR7] Chukmaitov AS, Bazzoli GJ, Harless DW, Hurley RE, Devers KJ, Zhao M (2009). Variations in inpatient mortality among hospitals in different system types, 1995 to 2000. Medical Care.

[CR8] Cummings JN, Cross R (2003). Structural properties of work groups and their consequences for performance. Social Networks.

[CR9] Fontanella, C., Guada, J., Phillips, G., Ranbom, L., & Fortney, J. (2014). *Individual and contextual-level factors associated with continuity of care for adults with schizophrenia*. Paper presented at the Adm Policy Ment Health. 10.1007/s10488-013-0500-x PMC3883973 23689992

[CR10] Haggerty JL, Reid RJ, Freeman GK, Starfield BH, Adair CE, McKendry R (2003). Continuity of care: A multidisciplinary review. British Medical Journal.

[CR11] Joyce AS, Adair CE, Wild TC, McDougall GM, Gordon A, Costigan N, Pasmeny G (2010). Continuity of care: Validation of a Self-report measure to assess client perceptions of mental health service delivery. Community Mental Health Journal.

[CR45] Knoke D, Yang S, Knoke D (2008). Social network analysis.

[CR12] Leavitt H (1951). Some effects of certain communication patterns on group performance. Journal of Abnormal and Social Psychology.

[CR13] Lehman AF, Postrado LT, Roth D, McNary SW, Goldman HH (1994). Continuity of care and client outcomes in the Robert Wood Johnson Foundation program on chronic mental illness. The Milbank Quarterly.

[CR14] Leutz WN (1999). Five Laws for integrating medical and social services: Lessons from the United States and the United Kingdom. The Milbank Quarterly.

[CR15] Leutz WN (1999). Five laws for integrating medical and social services: Lessons from the United States and the United Kingdom. Milbank Quarterly.

[CR16] Lorant V, Grard A, Nicaise P, Title 107 Study Group (2015). Implementing a nation-wide mental health care reform: An analysis of stakeholders’ priorities. Community Mental Health Journal.

[CR17] Malone D, Newron-Howes G, Simmonds S, Marriot S, Tyrer P (2007). Community mental health teams (CMHTs) for people with severe mental illnesses and disordered personality. Cochrane Database Systematics Reviews.

[CR18] Marwaha S, Durrani A, Singh S (2013). Employment outcomes in people with bipolar disorder: A systematic review. Acta Psychiatrica Scandinavica.

[CR19] Mascia D, Angeli F, Di Vincenzo F (2015). Effect of hospital referral networks on patient readmissions. Social Science and Medicine.

[CR20] McGuire M, Agranoff R (2011). The limitations of public management networks. Public Administration.

[CR21] Milward HB, Provan KG (1998). Measuring network structure. Public Administration.

[CR22] Mitchell SM, Shortell SM (2000). The governance and management of effective community health partnerships: A typology for research, policy, and practice. The Milbank Quarterly.

[CR23] Morrissey JP, Calloway M, Bartko WT, Ridgely MS, Goldman HH, Paulson RI (1994). Local mental health authorities and service system change: evidence from the Robert Wood Johnson program on chronic mental illness. The Milbank quarterly.

[CR24] Narrow WE, Regier DA, Norquist G, Rae DS, Kennedy C, Arons B (2000). Mental health service use by Americans with severe mental illnesses. Social Psychiatry and Psychiatric Epidemiology.

[CR25] Nicaise P, Dubois V, Lorant V (2014). Mental health care delivery system reform in Belgium: The challenge of achieving deinstitutionalisation whilst addressing fragmentation of care at the same time. Health Policy.

[CR26] Nicaise P, Tulloch S, Dubois V, Matanov A, Priebe S, Lorant V (2013). Using social network analysis for assessing mental health and social services inter-organisational collaboration: findings in deprived areas in Brussels and London. Administration and Policy in Mental Health.

[CR46] Oakes JM, Kaufman JS (2006). Methods in social epidemiology.

[CR27] OECD. (2013). Santé mentale et emploi. Paris: OECD Publishing.

[CR28] Pfiffner, C., Steinert, T., Kilian, R., Becker, T., Frasch, K., Eschweiler, G., Jaeger, S. (2014). *Rehospitalization risk of former voluntary and involuntary patients with schizophrenia*. Paper presented at the Soc Psychiatry Psychiatr Epidemiol. 10.1007/s00127-014-0892-2 24806950

[CR29] Priebe, S., Matanov, A., Barros, H., Canavan, R., Gabor, E., Greacen, T., Gaddini, A. (2012a). Mental health-care provision for marginalized groups across Europe: findings from the PROMO study. *The European Journal of Public Health*, ckr214. 10.1093/eurpub/ckr214 23132869

[CR30] Priebe S, Matanov A, Schor R, StraSZmayr C, Barros H, Barry M, Gaddini A (2012). Good practice in mental health care for socially marginalised groups in Europe: A qualitative study of expert views in 14 countries. BMC Public Health.

[CR31] Priebe S, Watzke S, Hansson L, Burns T (2008). Objective social outcomes index (SIX): A method to summarise objective indicators of social outcomes in mental health care. Acta Psychiatrica Scandinavica.

[CR32] Provan KG, Fish A, Sydow J (2007). Interorganizational networks at the network nevel: A review of the empirical literature on whole networks. Journal of Management.

[CR33] Provan KG, Milward HB (1995). A preliminary theory of interorganizational network effectiveness: A comparative study of four community mental health systems. Administrative Science Quarterly.

[CR34] Provan KG, Milward HB (2001). Do networks really work? A framework for evaluating public-sector organizational networks. Public Administration Review.

[CR35] Provan KG, Sebastian JG (1998). Networks within networks: Service link overlap, organizational cliques, and network effectiveness. The Academy of Management Journal.

[CR36] Provan KG, Veazie MA, Staten LK, Teufel-Shone NI (2005). The use of network analysis to strengthen community partnerships. Public Administration Review.

[CR37] Qin P, Nordentoft M (2005). Suicide risk in relation to psychiatric hospitalization: Evidence based on longitudinal registers. Archives of General Psychiatry.

[CR38] Rosenheck R, Morrissey J, Lam J, Calloway M, Johnsen M, Goldman H, Teague G (1998). Service system integration, access to services, and housing outcomes in a program for homeless persons with severe mental illness. American Journal of Public Health.

[CR39] Scharf DM, Eberhart NK, Schmidt N, Vaughan CA, Dutta T, Pincus HA, Burnam MA (2013). Integrating primary care into community behavioral health settings: Programs and early implementation experiences. Psychiatric Services.

[CR100] Schinnar AP (1990). An empirical literature review of definitions of severe and persistent mental illness. American Journal of Psychiatry.

[CR40] Shortell SM, Wu FM, Lewis VA, Colla CH, Fisher ES (2014). A taxonomy of accountable care organizations for policy and practice. Health Services Research.

[CR41] Shortell SM, Zukoski AP, Alexander JA, Bazzoli GJ, Conrad DA, Hasnain-Wynia R, Margolin FS (2002). Evaluating partnerships for community health improvement: Tracking the footprints. Journal of Health Politics, Policy and Law.

[CR42] Social psychiatry and psychiatric epidemiology. http://dx.doi.org/10.1007/s00127-014-0892-2.

[CR43] Turrini A, Cristofoli D, Frosini F, Nasi G (2010). Networking literature about determinants of network effectiveness. Public Administration.

[CR44] Wing JK, Beevor AS, Curtis RH, Park SB, Hadden S, Burns A (1998). Health of the Nation Outcome Scales (HoNOS). Research and development. The British Journal of Psychiatry.

