# Supplementary material for: Optimal Network for Patients with Severe Mental Illness: A Social Network Analysis
Source: Adm Policy Ment Health. 2017 Mar 24;44(6):877–87. doi: 10.1007/s10488-017-0800-7 (PMC5640746; doi:10.1007/s10488-017-0800-7)
Supplement: Supplementary file 1 — Supplementary material 1 (DOCX 15 KB) [file 10488_2017_800_MOESM1_ESM.docx]

Supplementary tables

| Supplementary Table 1. Alberta Continuity of Service Scale: mean and standard deviation per dimension and item. |  |  |
| --- | --- | --- |
| **Alberta Continuity of Service Scale: dimensions and items** | **Mean**  **(1 -5)** | **Std** |
| **Dimensions of the Alberta Continuity of Service Scale:** |  |  |
| Total score (/100) | 74.6 | 9.1 |
| System Fragmentation (/100) | 75.0 | 10.4 |
| Relationship base (/100) | 77.7 | 11.7 |
| Responsive treatment (/100) | 70.7 | 11.6 |
| **Items of the Alberta Continuity of Service Scale:** |  |  |
| I’ve had to repeat my history every time I needed help | 3.1 | 1.3 |
| My primary caregiver has called to check on me | 3.2 | 1.3 |
| My program is in touch with my family doctor/GP | 3.2 | 1.3 |
| If I run into problems I can get services even in the middle of the night | 3.3 | 1.2 |
| There is no single place to find out about all services available (§) | 3.4 | 1.2 |
| My records never seem to be available to new providers I see (§) | 3.4 | 1.1 |
| There don’t seem to be links from one service to the next (§) | 3.5 | 1.1 |
| I would be able to change providers if things don’t go well | 3.5 | 1.0 |
| I am reminded of appointments or called if I miss appointments | 3.5 | 1.2 |
| I do not feel involved in decisions about my care | 3.6 | 1.3 |
| I have to deal with a confusing number of programs (§) | 3.7 | 1.1 |
| I am not able to see my primary caregiver quickly when I need to (§) | 3.7 | 1.1 |
| Some services I need are just not out there (§) | 3.7 | 1.1 |
| My care doesn’t change when my needs change | 3.7 | 1.1 |
| I can easily get to the services I need | 3.8 | 0.9 |
| My appointments can be more frequent if I am doing worse | 3.8 | 1.2 |
| My primary caregiver asks me about more than just my symptoms | 3.8 | 1.2 |
| I have been able to get services in my own community | 3.8 | 1.1 |
| The longer I have been involved with services, the more satisfying my life has become | 3.9 | 0.9 |
| Those involved in my care don’t seem to talk with each other (§) | 3.9 | 1.1 |
| Everyone seems to work together for me | 3.9 | 0.9 |
| I was asked what I wanted out of my treatment | 3.9 | 1.1 |
| My care is checked regularly to see if it is working | 3.9 | 0.9 |
| I am not treated like an individual in mental health services (§) | 4.0 | 1.2 |
| I don’t know where I would go if I needed help | 4.0 | 1.1 |
| I’ve been refused admission to certain programs and was unable to understand why (§) | 4.0 | 1.1 |
| My treatment fits my needs | 4.0 | 0.9 |
| I’m confident that my psychiatrist can admit me whenever I need to be in the hospital (§) | 4.0 | 1.0 |
| I have been unable to pay for my medication when out of hospital (§) | 4.1 | 1.1 |
| My care team has encouraged me to take responsibility for my own care (§) | 4.1 | 0.8 |
| My provider knows about all the different services available | 4.2 | 0.8 |

§ Items with a negative wording. Coding has been reversed before computing the mean and std.

Supplementary Table 2. Mean network structure covariate by network cohort (n=19): means and t-test.

| **Network structure** | **Networks 2014** | **Networks 2012** | **t-test** | ***p* value** |
| --- | --- | --- | --- | --- |
| No. of networks | 9 | 10 |  |  |
| Degree centralization (%) | 19.6 | 23.8 | -0.6 | 0.57 |
| Coleman index prim. care (-1, 1) | -0.2 | -0.3 | 0.7 | 0.49 |
| Coleman index crisis/outreach teams | -0.5 | -0.2 | -2.1 | 0.05 |
| Coleman index social services | -0.3 | -0.1 | -1.4 | 0.17 |
| Density (%) | 15.9 | 16.8 | -0.3 | 0.75 |
| Index of dissimilarity (%) | 27.7 | 28.1 | -0.1 | 0.94 |
| Services (no.) | 32.5 | 59.9 | -2.3 | 0.03 |
| Social services (%) | 16.1 | 23.5 | -1.0 | 0.32 |
