# Supplementary material for: Optimal Network for Patients with Severe Mental Illness: A Social Network Analysis
Source: Adm Policy Ment Health. 2017 Mar 24;44(6):877–87. doi: 10.1007/s10488-017-0800-7 (PMC5640746; doi:10.1007/s10488-017-0800-7)
Supplement: Supplementary file 2 — Supplementary material 2 (PDF 3002 KB) [file 10488_2017_800_MOESM2_ESM.pdf]

	P6
Size	11
Density	0,74
All-deg centralization	3,22
Betw centralization	0,14

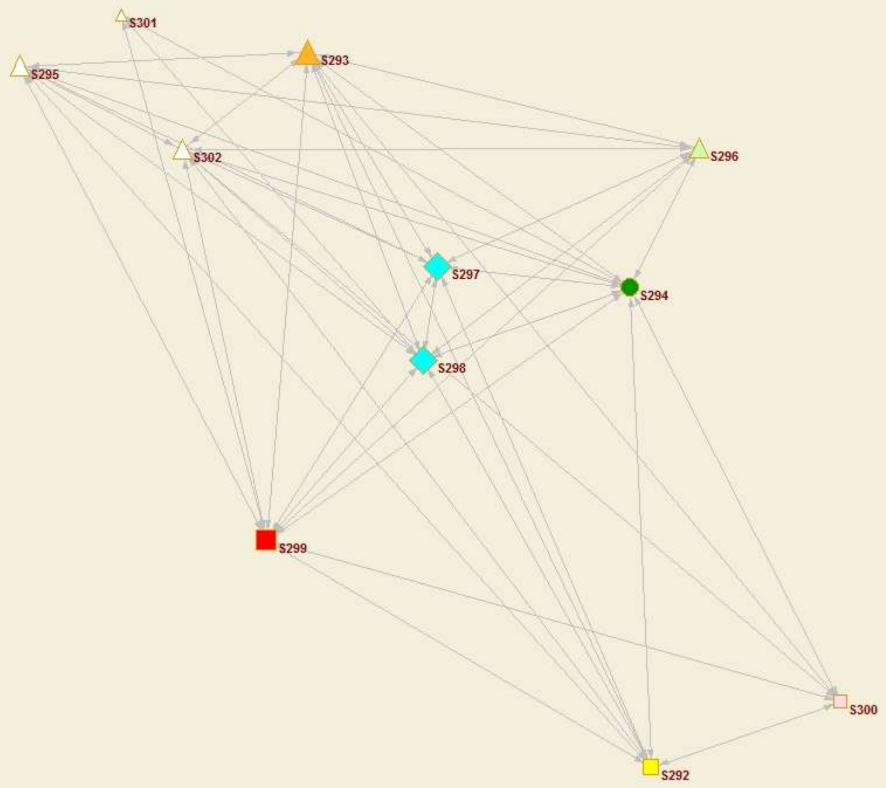

Service type	Symbol
Primary Care	
Community Mental Health	
Outreach Team	
Community rehabilitation	
Social service	
Ward in psychiatric hospital	
Psychiatric Ward General Hosp.	
Sheltered accommodation	
Psychiatric Nursing Home	
Self-Help	
Other	

	P19
Size	15
Density	0,65
All-deg centralization	5,69
Betw centralization	0,09

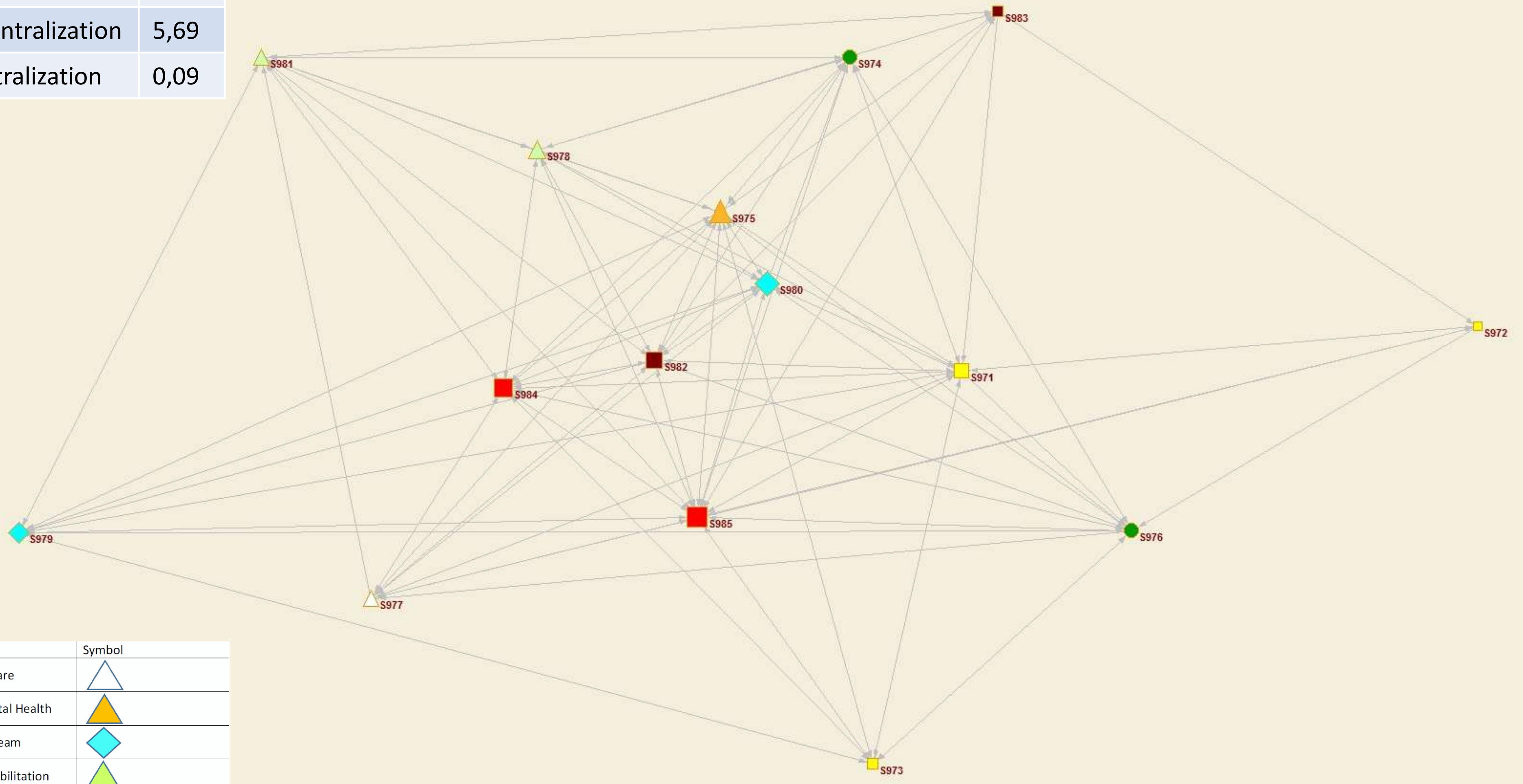

Service type	Symbol
Primary Care	
Community Mental Health	
Outreach Team	
Community rehabilitation	
Social service	
Ward in psychiatric hospital	
Psychiatric Ward General Hosp.	
Sheltered accommodation	
Psychiatric Nursing Home	
Self-Help	
Other	

	P3
Size	20
Density	0,63
All-deg centralization	6,72
Betw centralization	0,07

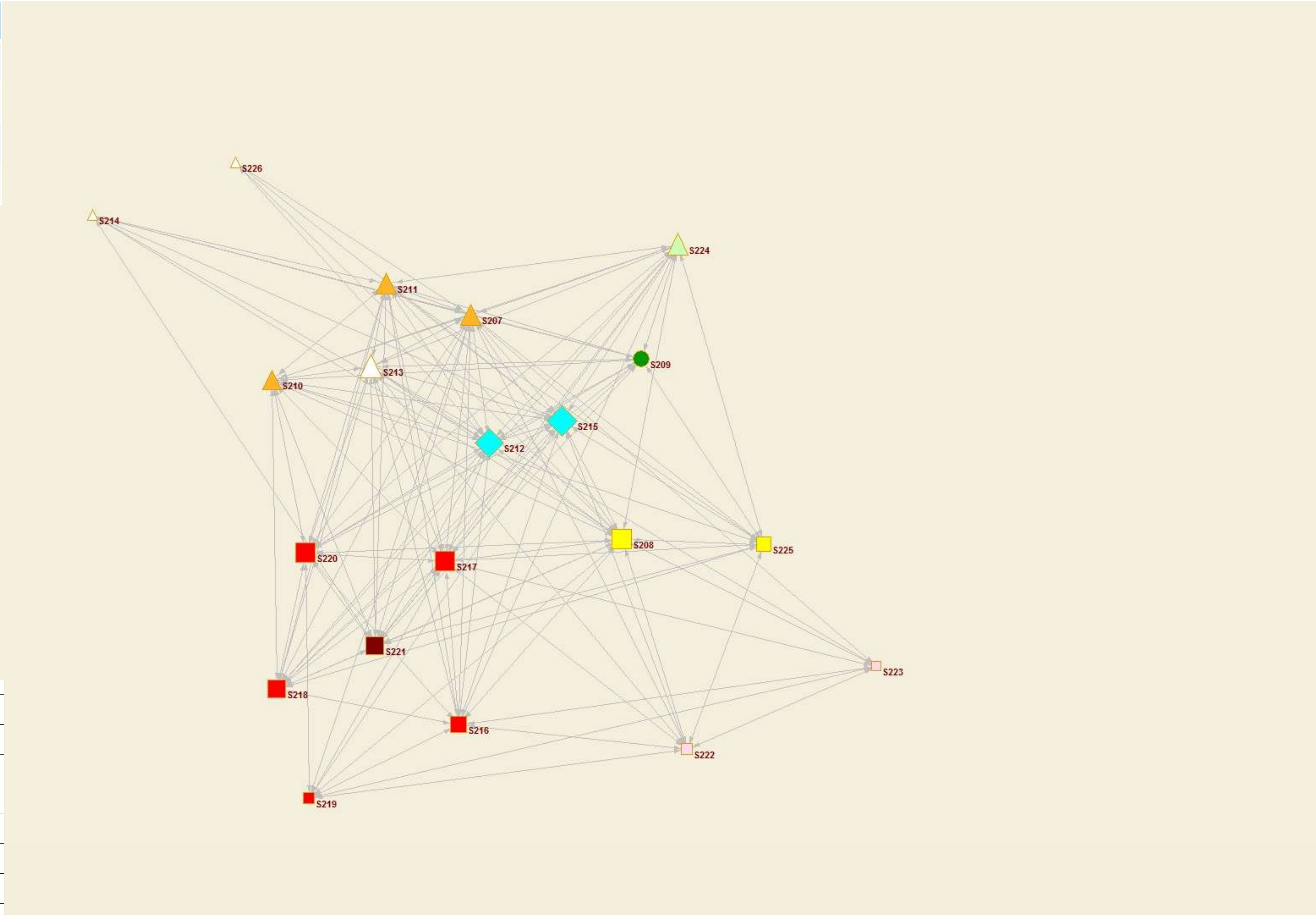

Service type	Symbol
Primary Care	
Community Mental Health	
Outreach Team	
Community rehabilitation	
Social service	
Ward in psychiatric hospital	
Psychiatric Ward General Hosp.	
Sheltered accommodation	
Psychiatric Nursing Home	
Self-Help	
Other	

	P8
Size	28
Density	0,67
All-deg centralization	9,15
Betw centralization	0,04

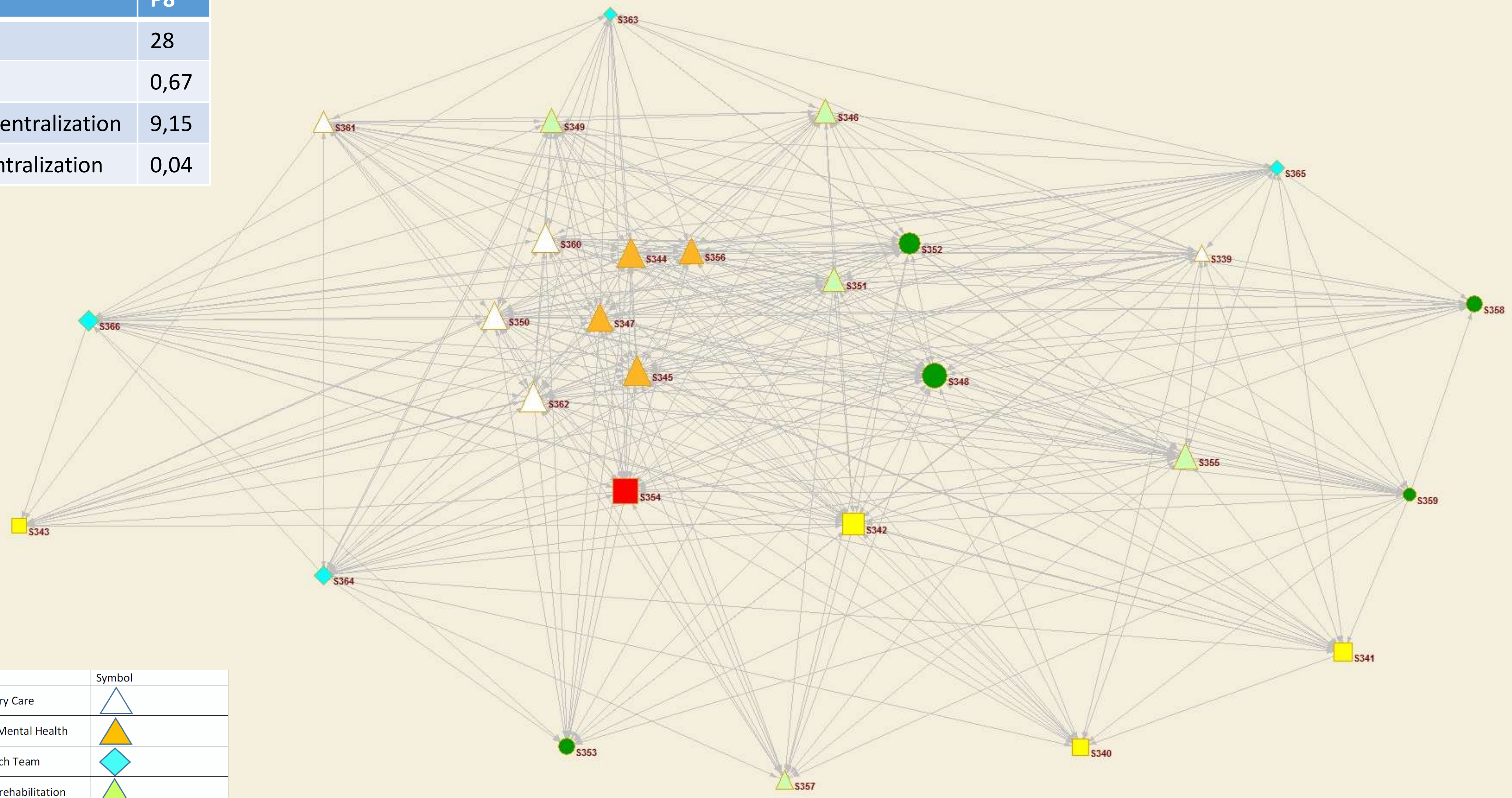

Service type	Symbol
Primary Care	
Community Mental Health	
Outreach Team	
Community rehabilitation	
Social service	
Ward in psychiatric hospital	
Psychiatric Ward General Hosp.	
Sheltered accommodation	
Psychiatric Nursing Home	
Self-Help	
Other	

	P4
Size	29
Density	0,21
All-deg centralization	19,87
Betw centralization	0,37

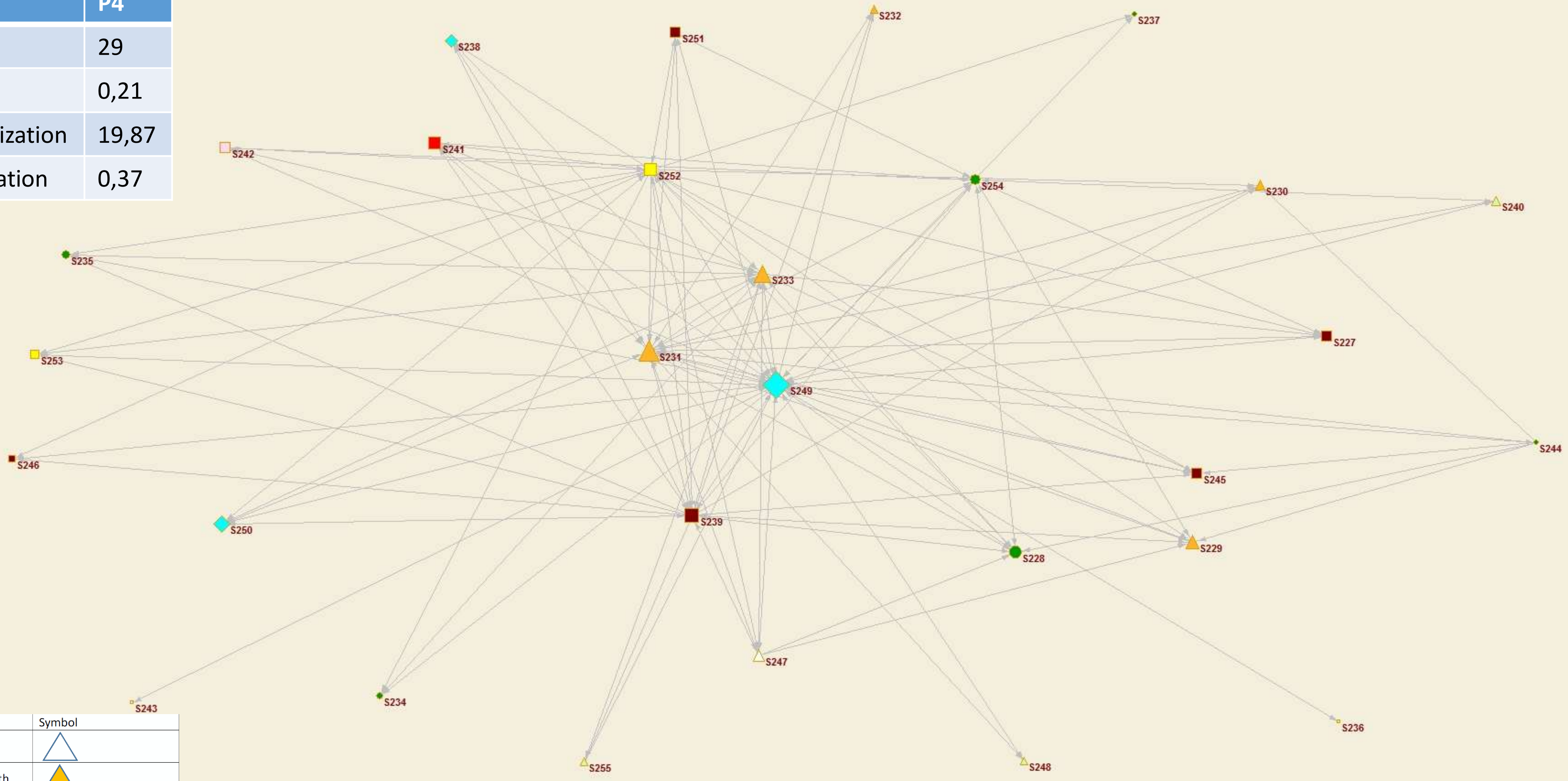

Service type	Symbol
Primary Care	
Community Mental Health	
Outreach Team	
Community rehabilitation	
Social service	
Ward in psychiatric hospital	
Psychiatric Ward General Hosp.	
Sheltered accommodation	
Psychiatric Nursing Home	
Self-Help	
Other	

	P16
Size	30
Density	0,57
All-deg centralization	13,50
Betw centralization	0,07

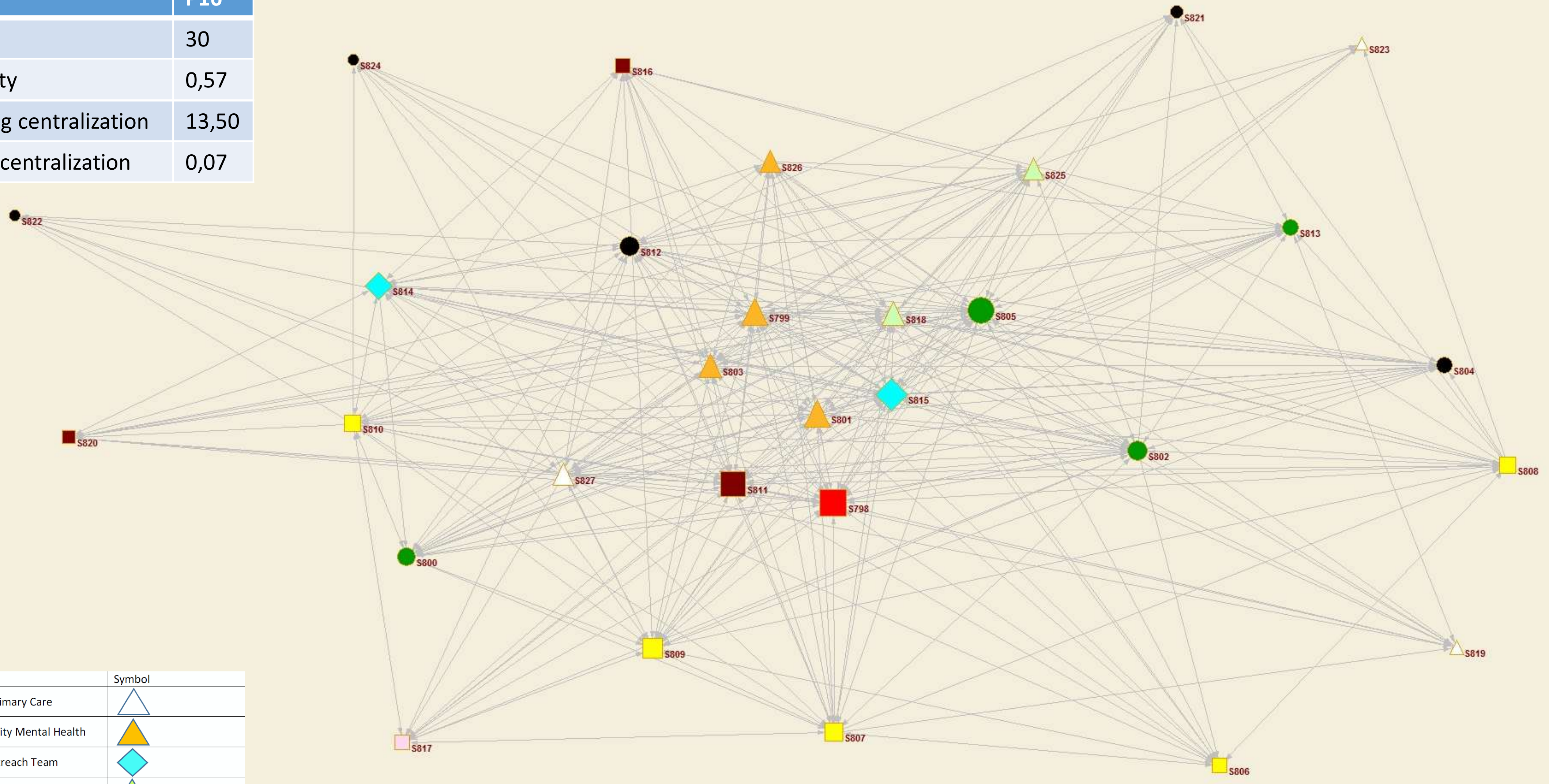

Service type	Symbol
Primary Care	
Community Mental Health	
Outreach Team	
Community rehabilitation	
Social service	
Ward in psychiatric hospital	
Psychiatric Ward General Hosp.	
Sheltered accommodation	
Psychiatric Nursing Home	
Self-Help	
Other	

	P11
Size	33
Density	0,37
All-deg centralization	18,42
Betw centralization	0,14

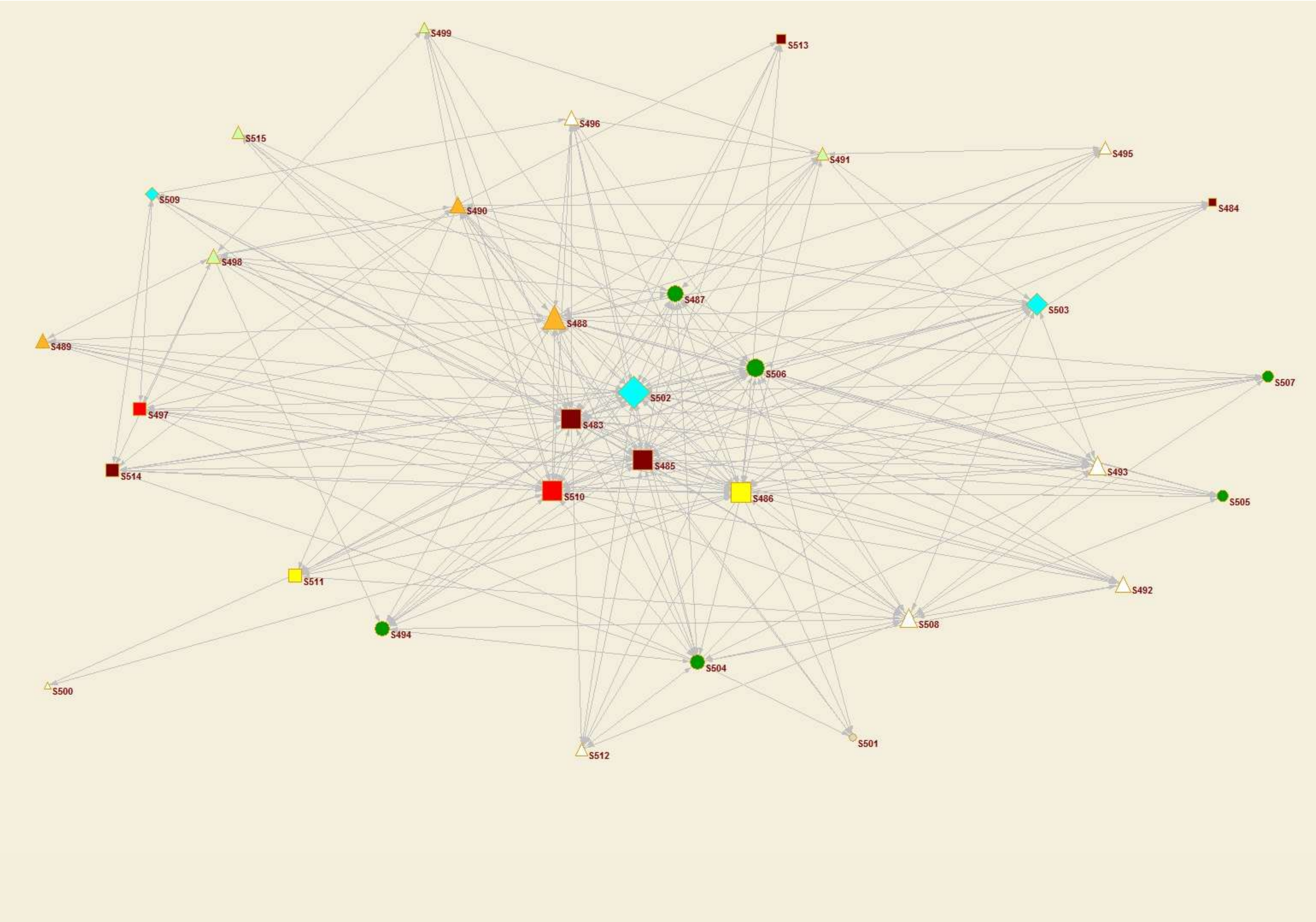

Service type	Symbol
Primary Care	
Community Mental Health	
Outreach Team	
Community rehabilitation	
Social service	
Ward in psychiatric hospital	
Psychiatric Ward General Hosp.	
Sheltered accommodation	
Psychiatric Nursing Home	
Self-Help	
Other	

	P7
Size	36
Density	0,40
All-deg centralization	16,97
Betw centralization	0,09

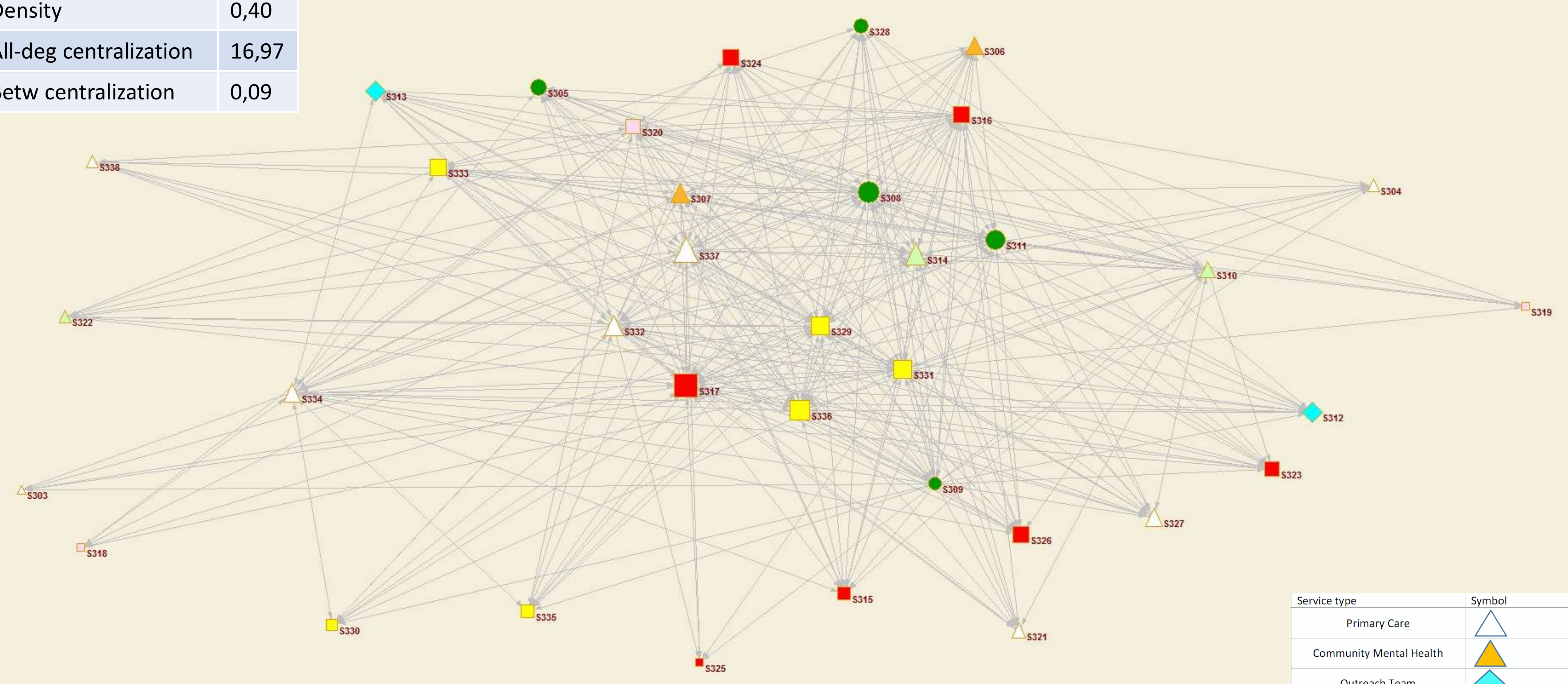

Service type	Symbol
Primary Care	
Community Mental Health	
Outreach Team	
Community rehabilitation	
Social service	
Ward in psychiatric hospital	
Psychiatric Ward General Hosp.	
Sheltered accommodation	
Psychiatric Nursing Home	
Self-Help	
Other	

	P5
Size	36
Density	0,35
All-deg centralization	22,06
Betw centralization	0,26

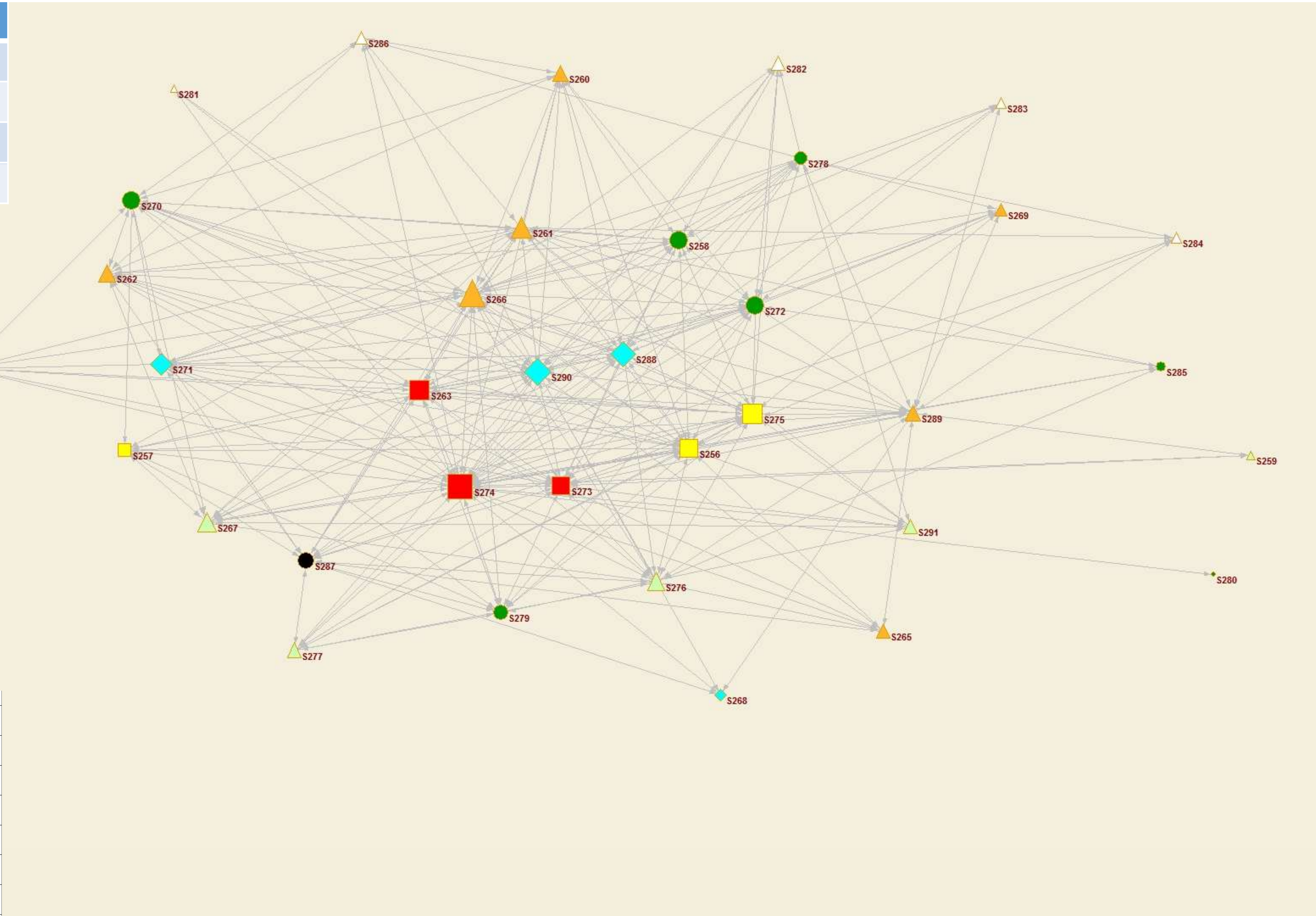

Service type	Symbol
Primary Care	
Community Mental Health	
Outreach Team	
Community rehabilitation	
Social service	
Ward in psychiatric hospital	
Psychiatric Ward General Hosp.	
Sheltered accommodation	
Psychiatric Nursing Home	
Self-Help	
Other	

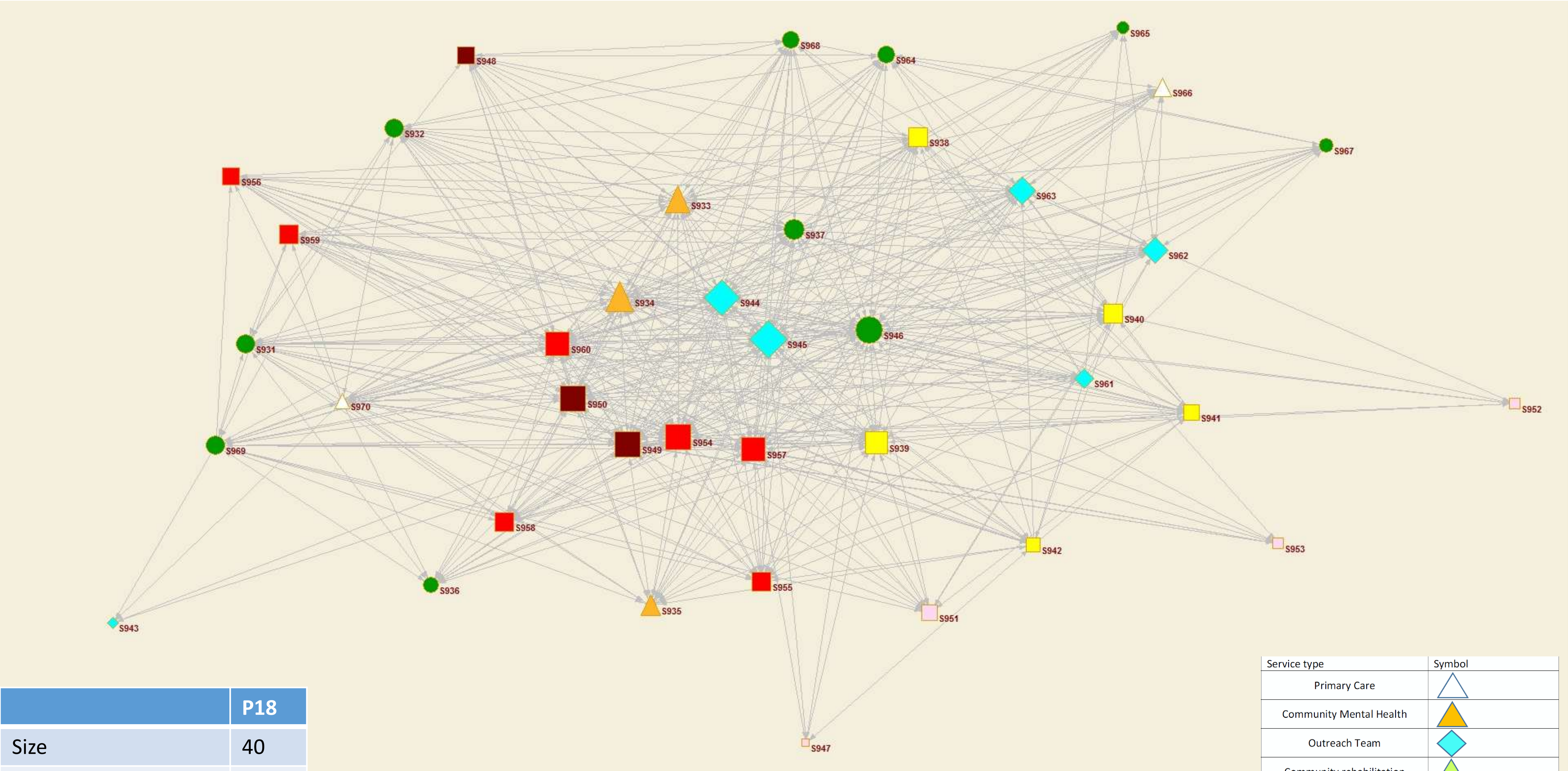

	P18
Size	40
Density	0,50
All-deg centralization	20,42
Betw centralization	0,10

Service type	Symbol
Primary Care	
Community Mental Health	
Outreach Team	
Community rehabilitation	
Social service	
Ward in psychiatric hospital	
Psychiatric Ward General Hosp.	
Sheltered accommodation	
Psychiatric Nursing Home	
Self-Help	
Other	

	P9
Size	41
Density	0,41
All-deg centralization	24,64
Betw centralization	0,11

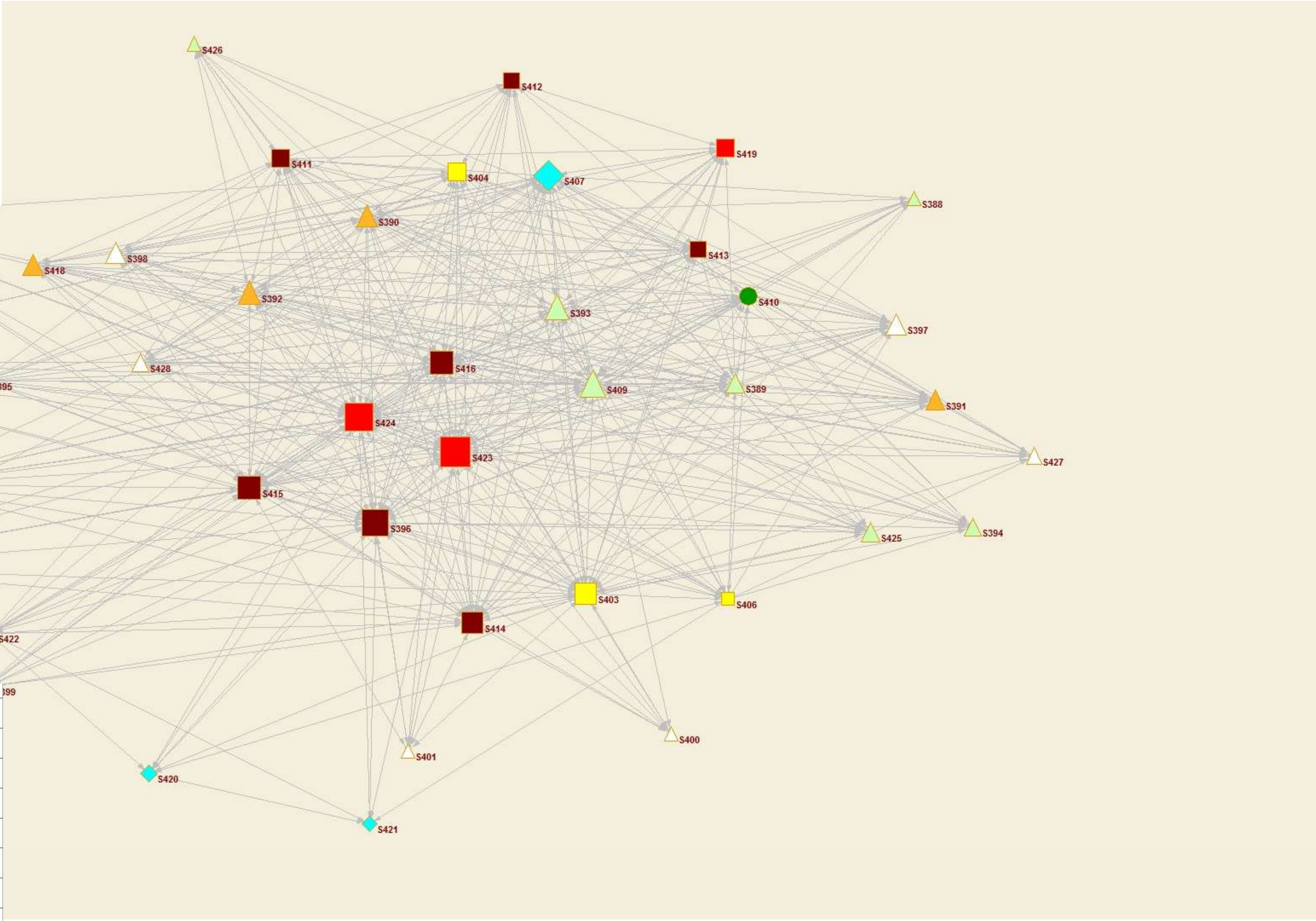

Service type	Symbol
Primary Care	
Community Mental Health	
Outreach Team	
Community rehabilitation	
Social service	
Ward in psychiatric hospital	
Psychiatric Ward General Hosp.	
Sheltered accommodation	
Psychiatric Nursing Home	
Self-Help	
Other	

	P10
Size	54
Density	0,58
All-deg centralization	22,06
Betw centralization	0,03

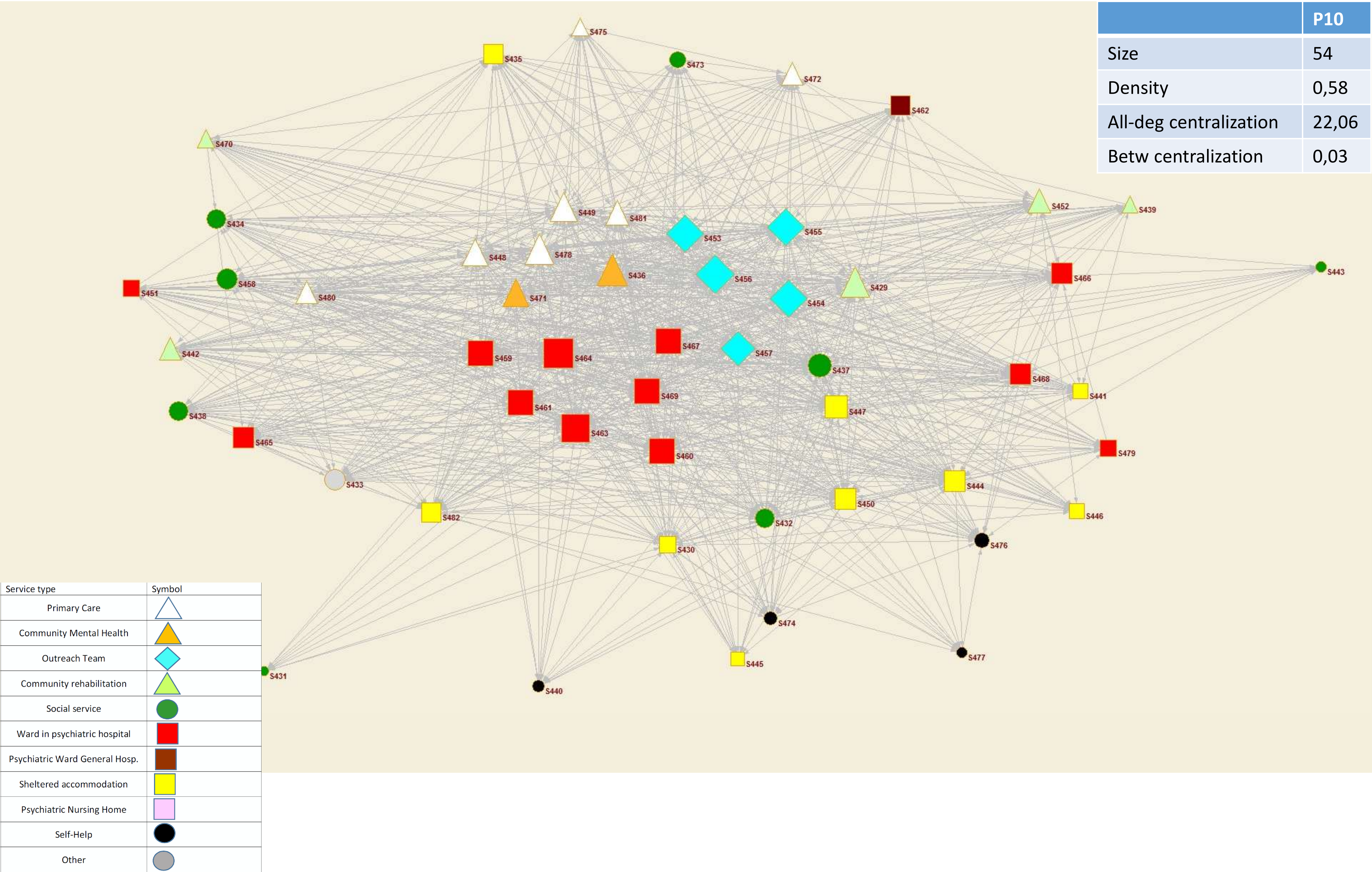

Service type	Symbol
Primary Care	
Community Mental Health	
Outreach Team	
Community rehabilitation	
Social service	
Ward in psychiatric hospital	
Psychiatric Ward General Hosp.	
Sheltered accommodation	
Psychiatric Nursing Home	
Self-Help	
Other	

	P12
Size	60
Density	0,23
All-deg centralization	33,12
Betw centralization	0,16

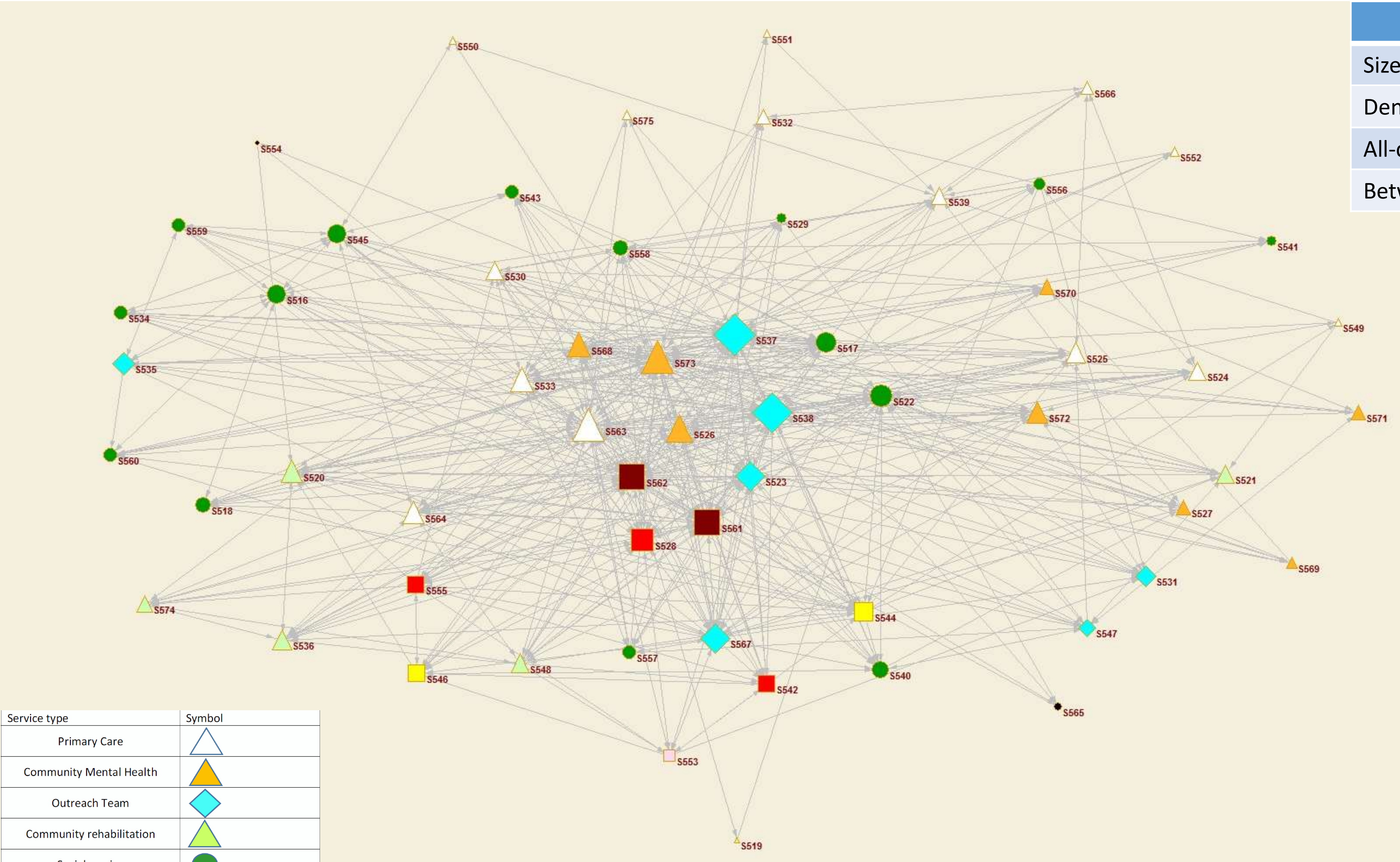

Service type	Symbol
Primary Care	
Community Mental Health	
Outreach Team	
Community rehabilitation	
Social service	
Ward in psychiatric hospital	
Psychiatric Ward General Hosp.	
Sheltered accommodation	
Psychiatric Nursing Home	
Self-Help	
Other	

	P14
Size	67
Density	0,31
All-deg centralization	36,85
Betw centralization	0,08

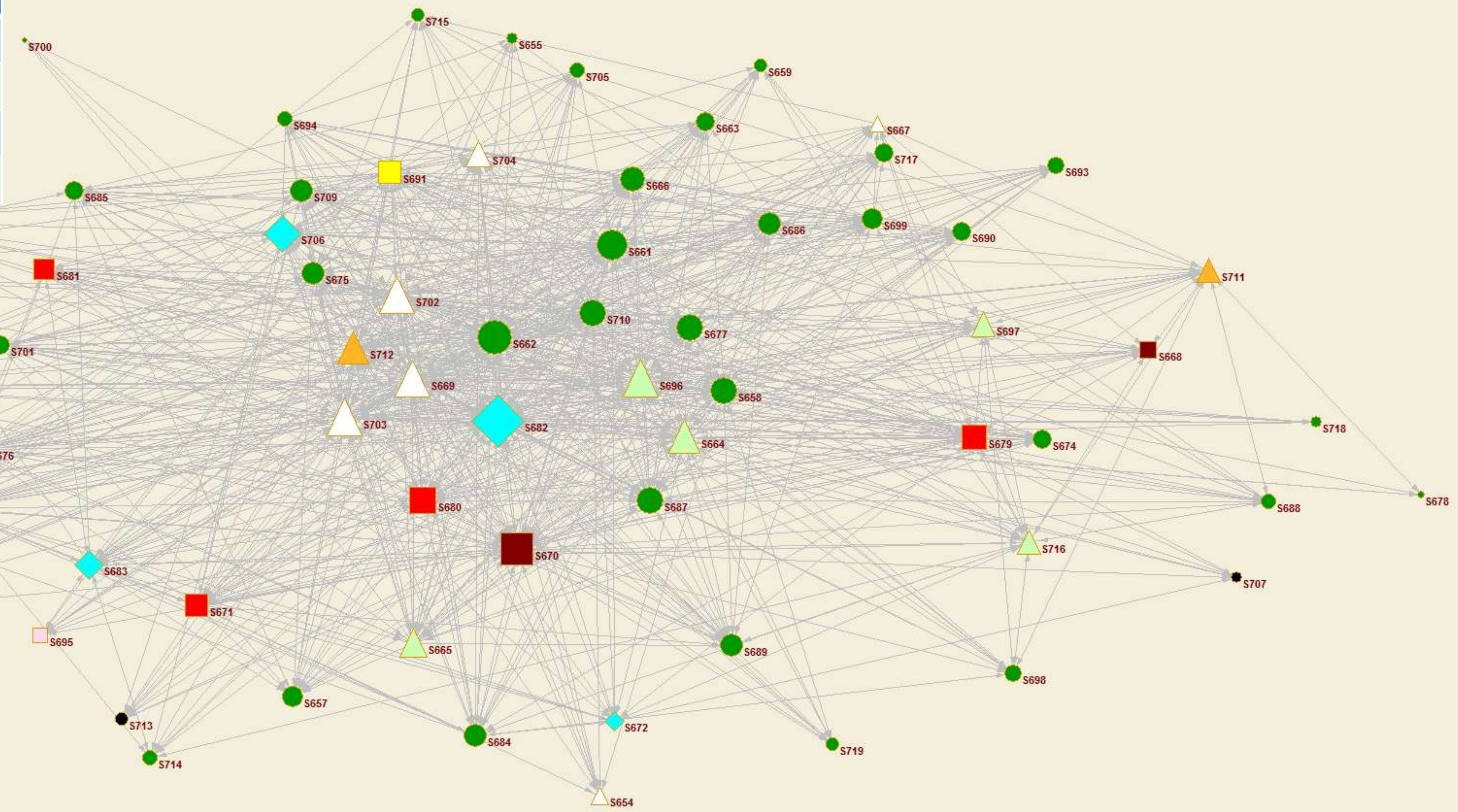

Service type	Symbol
Primary Care	
Community Mental Health	
Outreach Team	
Community rehabilitation	
Social service	
Ward in psychiatric hospital	
Psychiatric Ward General Hosp.	
Sheltered accommodation	
Psychiatric Nursing Home	
Self-Help	
Other	

	P13
Size	77
Density	0,17
All-deg centralization	44,45
Betw centralization	0,23

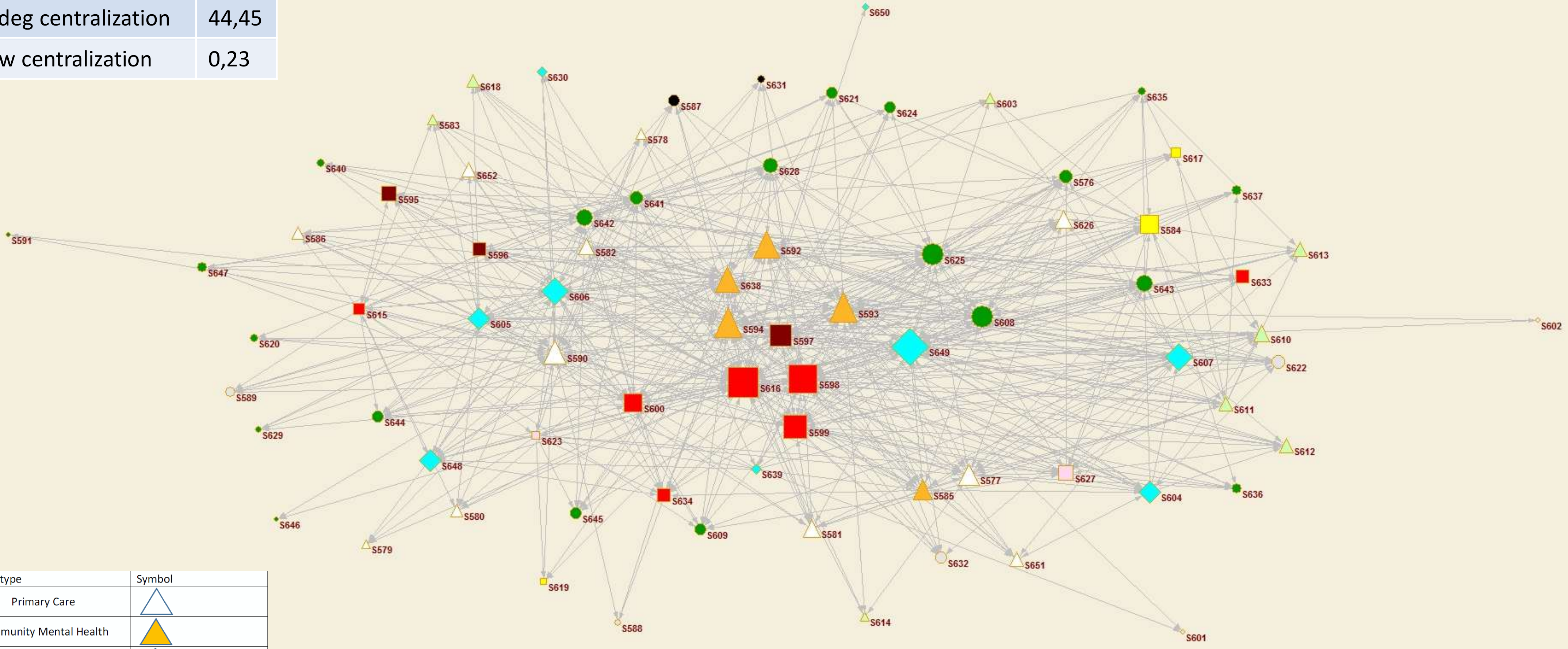

Service type	Symbol
Primary Care	
Community Mental Health	
Outreach Team	
Community rehabilitation	
Social service	
Ward in psychiatric hospital	
Psychiatric Ward General Hosp.	
Sheltered accommodation	
Psychiatric Nursing Home	
Self-Help	
Other	

	P15
Size	78
Density	0,25
All-deg centralization	50,84
Betw centralization	0,15

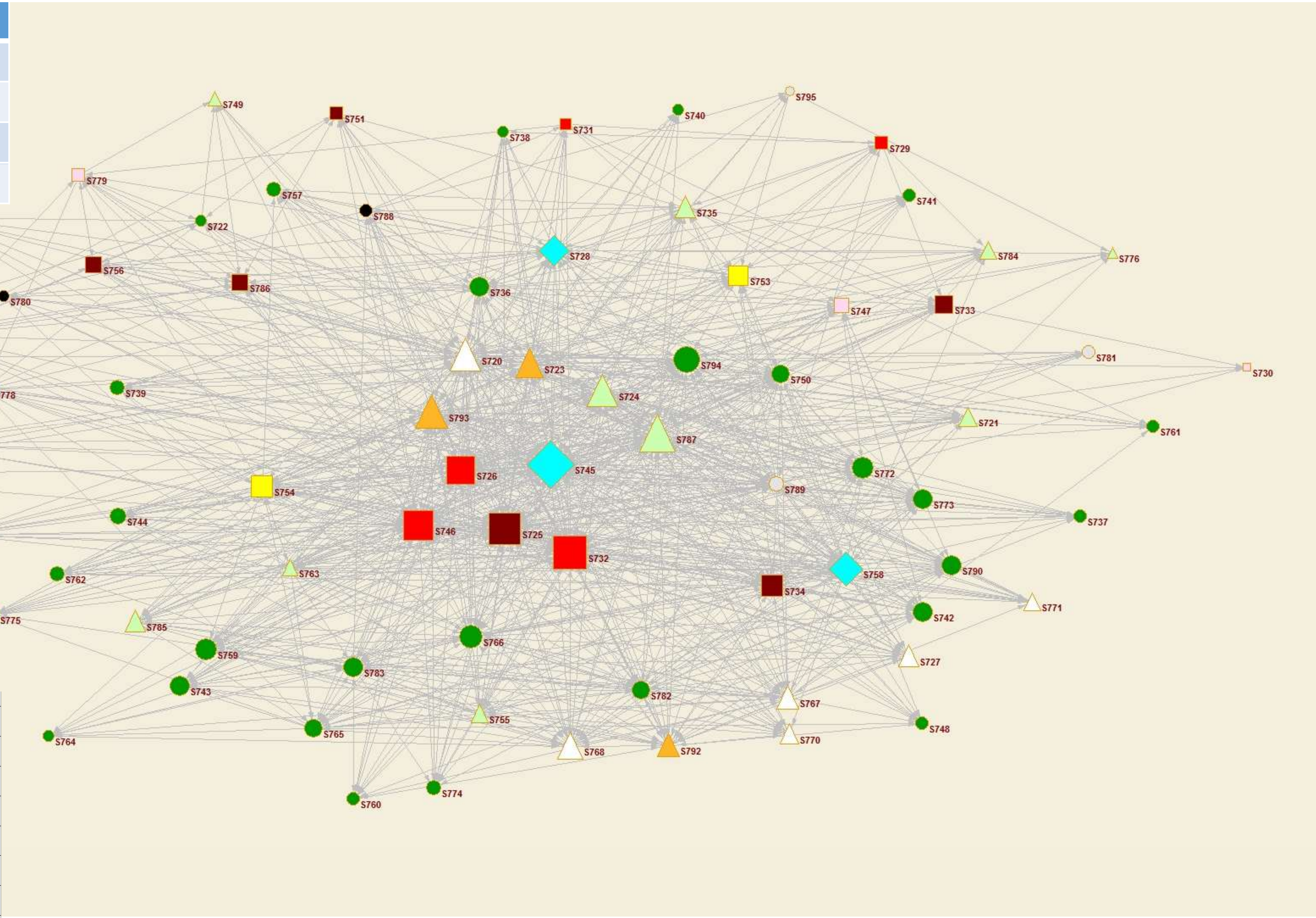

Service type	Symbol
Primary Care	
Community Mental Health	
Outreach Team	
Community rehabilitation	
Social service	
Ward in psychiatric hospital	
Psychiatric Ward General Hosp.	
Sheltered accommodation	
Psychiatric Nursing Home	
Self-Help	
Other	

	P1
Size	101
Density	0,17
All-deg centralization	72,39
Betw centralization	0,14

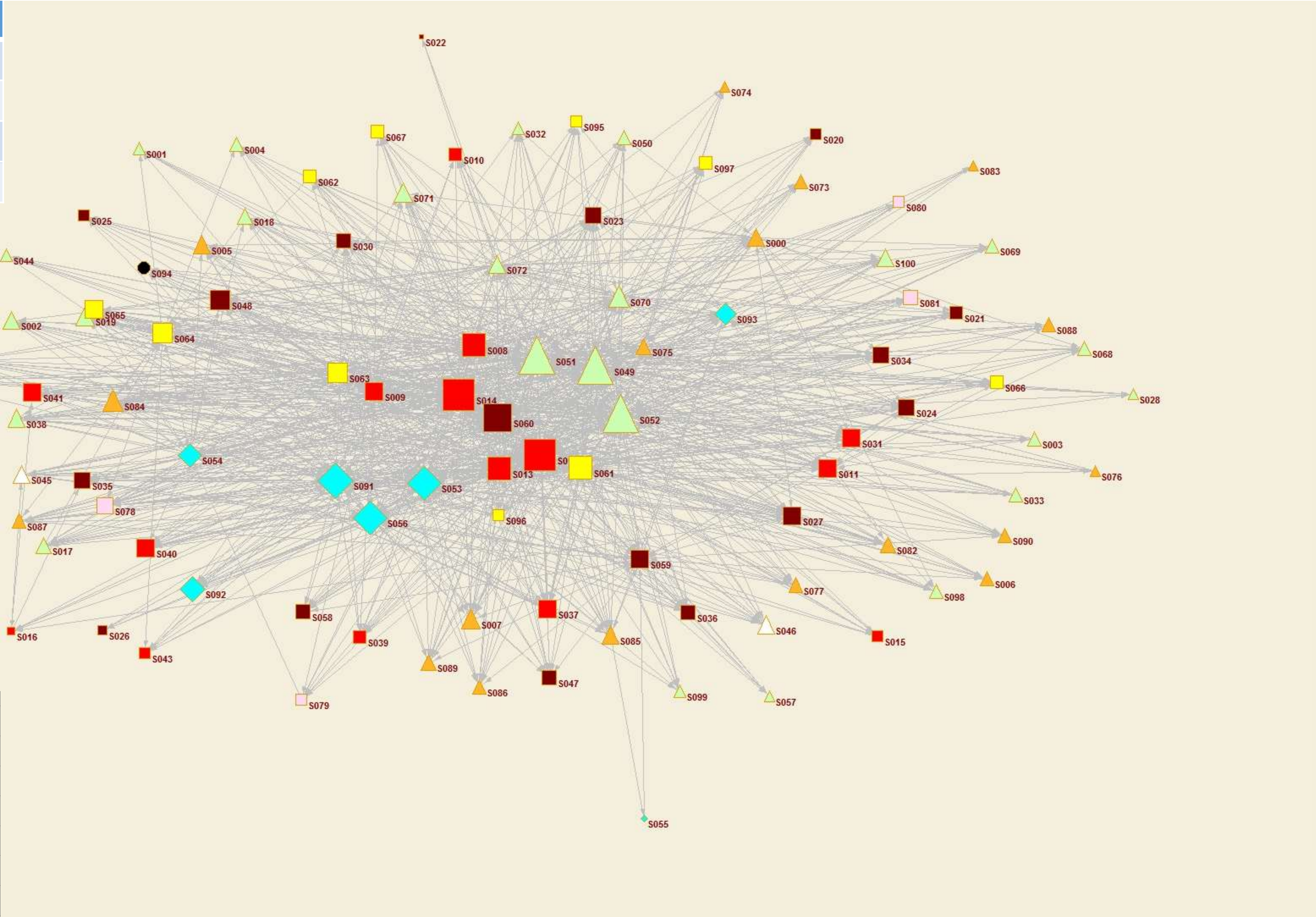

Service type	Symbol
Primary Care	
Community Mental Health	
Outreach Team	
Community rehabilitation	
Social service	
Ward in psychiatric hospital	
Psychiatric Ward General Hosp.	
Sheltered accommodation	
Psychiatric Nursing Home	
Self-Help	
Other	

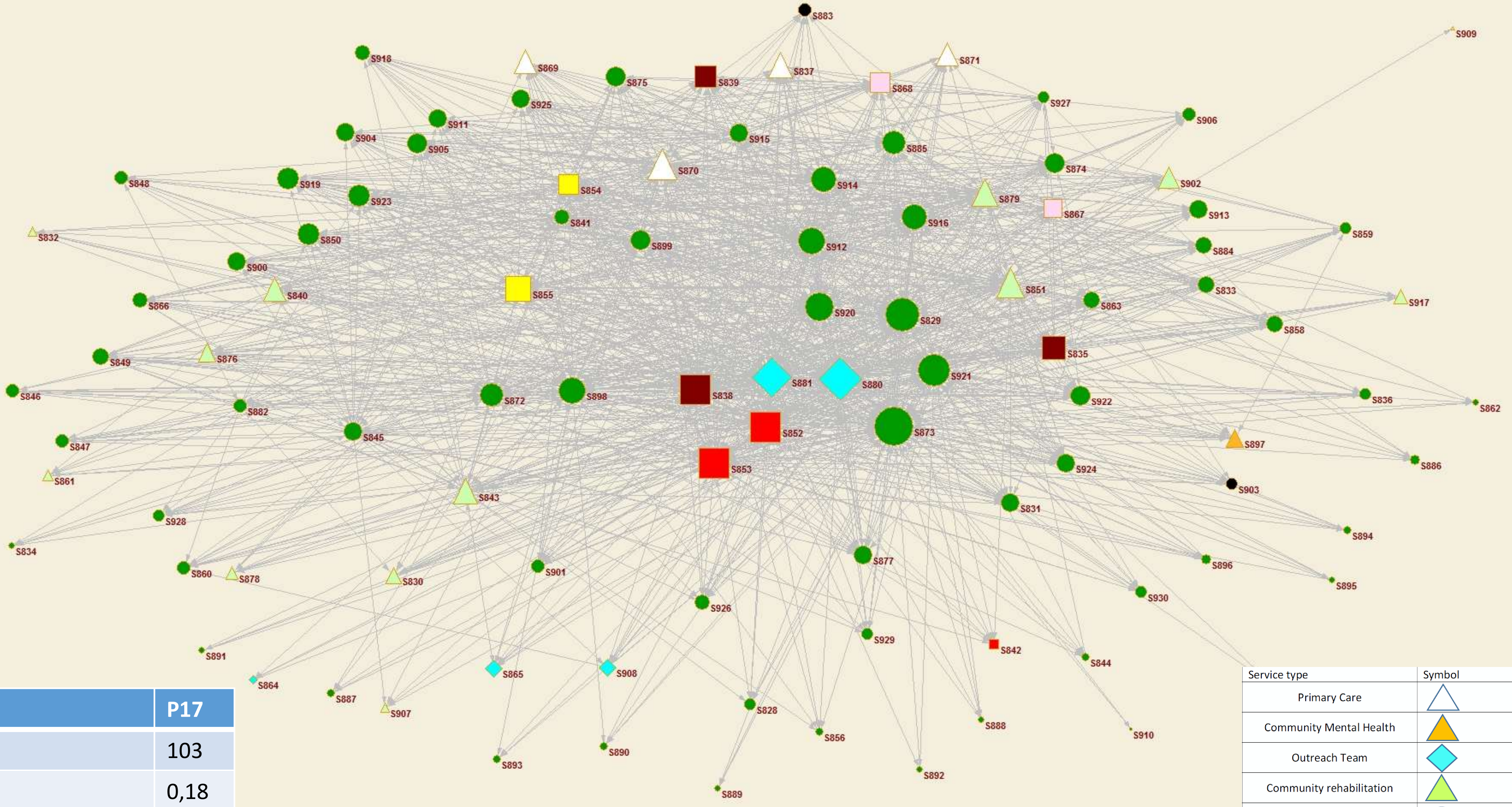

	P17
Size	103
Density	0,18
All-deg centralization	66,98
Betw centralization	0,18

Service type	Symbol
Primary Care	
Community Mental Health	
Outreach Team	
Community rehabilitation	
Social service	
Ward in psychiatric hospital	
Psychiatric Ward General Hosp.	
Sheltered accommodation	
Psychiatric Nursing Home	
Self-Help	
Other	

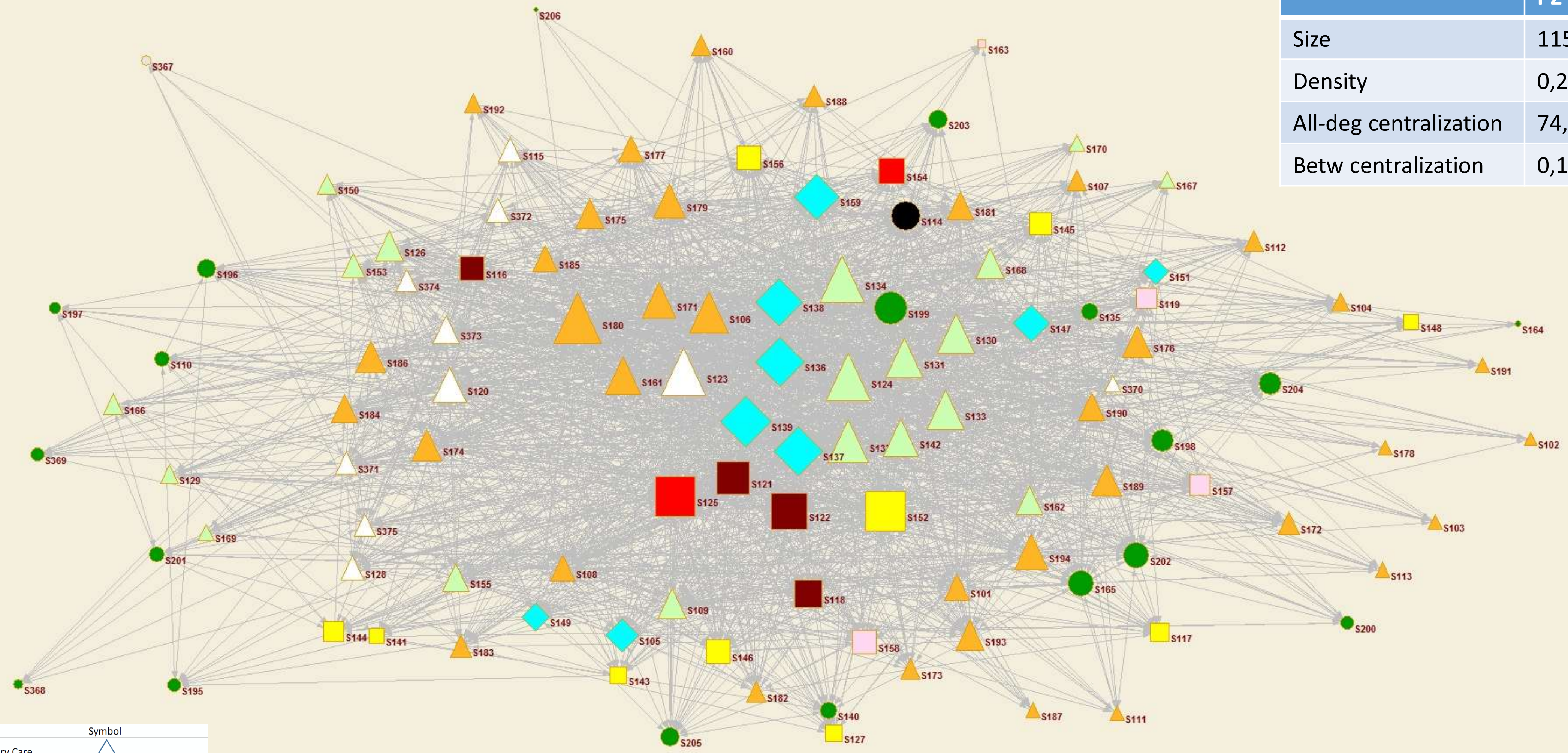

	P2
Size	115
Density	0,26
All-deg centralization	74,15
Betw centralization	0,13

Service type	Symbol
Primary Care	
Community Mental Health	
Outreach Team	
Community rehabilitation	
Social service	
Ward in psychiatric hospital	
Psychiatric Ward General Hosp.	
Sheltered accommodation	
Psychiatric Nursing Home	
Self-Help	
Other	
